# *Echinococcus granulosus* Antigen B binds to monocytes and macrophages modulating cell response to inflammation

**DOI:** 10.1186/s13071-016-1350-7

**Published:** 2016-02-04

**Authors:** Valeria Silva-Álvarez, Ana Maite Folle, Ana Lía Ramos, Eduardo S. Kitano, Leo K. Iwai, Inés Corraliza, Betina Córsico, Ana María Ferreira

**Affiliations:** Cátedra de Inmunología, Facultad de Ciencias/Facultad de Química, Universidad de la República (UdelaR), Montevideo, Uruguay; Instituto de Investigaciones Bioquímicas de La Plata (INIBIOLP), Facultad de Ciencias Médicas, Universidad Nacional de La Plata (UNLP), La Plata, Argentina; Consejo Nacional de Investigaciones Científicas y Técnicas (CONICET), Ciudad Autónoma de Buenos Aires, Argentina; Laboratório Especial de Toxinologia Aplicada, Center of Toxins, Immune-Response and Cell Signalling (CeTICS), Instituto Butantan, São Paulo, Brazil; Departamento de Bioquímica, Biología Molecular y Genética, Facultad de Veterinaria, Universidad de Extremadura (UNEX), Cáceres, España

**Keywords:** Echinococcus granulosus, Antigen B, HLBP, Cell binding, Lipoproteins, Monocytes, Macrophages

## Abstract

**Background:**

Antigen B (EgAgB) is an abundant lipoprotein released by the larva of the cestode *Echinococcus granulosus* into the host tissues. Its protein moiety belongs to the cestode-specific family known as hydrophobic ligand binding protein (HLBP), and is encoded by five gene subfamilies (EgAgB8/1-EgAgB8/5). The functions of EgAgB in parasite biology remain unclear. It may play a role in the parasite’s lipid metabolism since it carries host lipids that *E. granulosus* is unable to synthesise. On the other hand, there is evidence supporting immuno-modulating activities in EgAgB, particularly on innate immune cells. Both hypothetical functions might involve EgAgB interactions with monocytes and macrophages, which have not been formally analysed yet.

**Methods:**

EgAgB binding to monocytes and macrophages was studied by flow cytometry using inflammation-recruited peritoneal cells and the THP-1 cell line. Involvement of the protein and phospholipid moieties in EgAgB binding to cells was analysed employing lipid-free recombinant EgAgB subunits and phospholipase D treated-EgAgB (lacking the polar head of phospholipids). Competition binding assays with plasma lipoproteins and ligands for lipoprotein receptors were performed to gain information about the putative EgAgB receptor(s) in these cells. Arginase-I induction and PMA/LPS-triggered IL-1β, TNF-α and IL-10 secretion were examined to investigate the outcome of EgAgB binding on macrophage response.

**Results:**

Monocytes and macrophages bound native EgAgB specifically; this binding was also found with lipid-free rEgAgB8/1 and rEgAgB8/3, but not rEgAgB8/2 subunits. EgAgB phospholipase D-treatment, but not the competition with phospholipid vesicles, caused a strong inhibition of EgAgB binding activity, suggesting an indirect contribution of phospholipids to EgAgB-cell interaction. Furthermore, competition binding assays indicated that this interaction may involve receptors with affinity for plasma lipoproteins. At functional level, the exposure of macrophages to EgAgB induced a very modest arginase-I response and inhibited PMA/LPS-mediated IL-1β and TNF-α secretion in an IL-10-independent manner.

**Conclusion:**

EgAgB and, particularly its predominant EgAgB8/1 apolipoprotein, are potential ligands for monocyte and macrophage receptors. These receptors may also be involved in plasma lipoprotein recognition and induce an anti-inflammatory phenotype in macrophages upon recognition of EgAgB.

**Electronic supplementary material:**

The online version of this article (doi:10.1186/s13071-016-1350-7) contains supplementary material, which is available to authorized users.

## Background

Cystic echinococcosis is a worldwide zoonotic infection caused by the larval stage of *Echinococcus granulosus* sensu lato species complex, which includes at least seven species that affects humans and livestock with significant economic and public health impact [1–5]. The larvae (metacestodes) of these species are fluid-filled, bladder-like structures that establish and gradually grow in the parenchyma of host internal organs, most commonly liver and lungs. They are usually called hydatid cysts, although strictly the term cyst includes a fibrous adventitial layer generated as a consequence of the host inflammatory reaction. Once the larva matures and reaches fertility, it generates protoscolex (PE), which are the parasite forms capable of developing into the adult worm in the definitive host (usually dogs). The fluid contained within the cyst, known as hydatid cyst fluid (HF), collects a variety of products excreted or secreted by the cellular, germinal layer (GL) of the cyst wall, as well as by protoscoleces. In addition, HF collects a variety of host plasma proteins (mostly albumin and immunoglobulins) which cross the cyst wall by unidentified mechanisms. This work refers to *E. granulosus* sensu lato, but for simplicity we will use the term *E. granulosus*.

One of the major HF components synthesised in abundance by the larva is a lipoprotein named Antigen B (EgAgB) [6]. The composition and antigenicity of EgAgB has been extensively studied, due to the fact that it constitutes the most immunogenic and specific *Echinococcus*-genus antigen for human serodiagnosis [7–10]. Molecular studies for characterising EgAgB protein moiety showed that it is encoded by a polymorphic and multigenic family that comprises five subfamilies named *EgAgB1* to *EgAgB5* [11–17]. These genes are differentially expressed in single lifecycle stages of the parasite, as well as within distinct tissues of a given developmental stage. *EgAgB1* to *EgAgB4* are expressed in the metacestode stage whereas *EgAgB5* seems to be mostly expressed in the adult stage. Regarding the metacestode, the expression of all genes was detected in GL, being *EgAgB1* and *EgAgB3* the most abundant, while in PE *EgAgB3* seems to be over-represented [18].

The mature protein products of EgAgB genes are α-helix rich polypeptides of 8 kDa, referred to as EgAgB8/1 to EgAgB8/5 subunits or apolipoproteins. Interestingly, EgAgB was found to belong to a novel cestode-specific family known as *Hydrophobic Ligand Binding Proteins* (HLBPs) [19], which have emerged by independent gene expansion events in different species [18]. More recently, our group has made substantial progress in the biochemical characterisation of EgAgB by developing novel methodological tools for purifying and characterising the lipid-free EgAgB8 apolipoproteins [20], and by determining which are its native lipid components [21]. We showed that in vitro lipid-free EgAgB8 subunits oligomerise; which agrees with their electrostatic profile predicted by structure modelling [22, 23]. They bound lipids selectively, particularly phospholipids and fatty acids rather than cholesterol [20], confirming previous observations [19]. Binding of lipids may enhance the oligomerisation of EgAgB8 subunits, favouring the formation of large lipoprotein complexes. We showed that the native antigen is a large (about 230 kDa in mass) lipoprotein particle, in which lipids account for about one-half of EgAgB total mass, comprising a heterogeneous mixture of neutral (mainly triacylglycerides, sterols and sterol esters) and polar lipids (mainly phosphatidylcholine) [21]. In sum, EgAgB may adopt a structural organisation similar to that of vertebrate high density lipoprotein (HDL), in which around a dozen EgAgB8 apolipoproteins would be embedded in an outer, hydrophilic phospholipid layer that surrounds the hydrophobic core of the lipoprotein particle [23].

We have thus achieved a better knowledge of EgAgB chemical composition and physicochemical properties. However, the understanding of the role of EgAgB in the parasite adaptation to host environment is still very rudimentary. It is uncertain how and when EgAgB is transported outside of the hydatid cyst; its apparent absence in the laminar layer as well as in the apical side of the tegumental syncytium of the GL does not support the existence of active secretion mechanisms in GL for this transport [24, 25]. Nevertheless, the fact that the host mounts a strong specific antibody response against EgAgB reveals that it reaches host tissues. Furthermore, an EgAgB ortholog in *Taenia solium* (TsM 150 kDa HLBP) was localised within the granuloma, adjacent to host parenchyma cells [26]. EgAgB contains lipids that *E. granulosus* is not able to synthesise [27, 28], such as fatty acids and cholesterol, and this supports the idea that EgAgB participates in parasite’s lipid metabolism (reviewed by [23]). Alternatively, EgAgB effects on various innate immune cells, including neutrophils, monocytes and dendritic cells [29–32], have led to the concept that this antigen is capable of modulating local inflammation and the subsequent mechanisms that activate T lymphocytes, favouring the generation of a Th2-type specific response [31]. Nevertheless, this Th2-biasing activity needs to be verified using the native lipoprotein, since it was described using denatured EgAgB (purified using electroelution [28] or after heating at 100 °C [31]). In any case, a putative EgAgB role in parasite mechanisms associated with lipid metabolism or immunomodulation would involve a direct interaction between EgAgB and host cells, likely mediated by cell receptors, and these interactions have not been examined yet.

In this work, we studied the ability of monocytes and macrophages to bind EgAgB selectively as well as the impact of EgAgB binding on the activation phenotype of macrophages. Our results show that EgAgB, particularly its apolipoprotein component, binds to monocytes and macrophages specifically, using receptors shared with plasma lipoproteins. Furthermore, EgAgB receptors in macrophages seem to induce signalling events involved in the regulation of inflammatory pathways.

## Methods

### Reagents

Antibiotic solutions, 3,5-di-tert-butyl–4-hydroxytoluene (BHT), cell culture reagents, dimethyl sulfoxide (DMSO), inorganic salts, ovalbumin (OVA), L-arginine, lipopolysaccharides (LPS) from *Escherichia coli*, phorbol 12-myristate 13-acetate (PMA), reduced glutathione, RPMI 1640 culture medium, streptavidin-peroxidase, 3,3′,5,5′-tetramethylbenzidine (TMB), thrombin from human plasma and urea were acquired from Sigma Chemicals (USA). Ethylenediaminetetraacetic acid (EDTA) and potassium bromide were obtained from Applichem (Germany). Acetonitrile was purchased from JT Baker (USA) and trifluoroacetic acid from Merck (Germany). Gluthathione Sepharose 4B and Q-Sepharose resins were acquired from GE Healthcare Life Sciences (Sweden). C8-bonded silica column was purchased from Vydac (USA). Human monocyte-like THP-1 cell line was obtained from American Type Culture Collection (ATCC, USA). Fetal bovine serum (FS) was purchased from Gibco (USA) and N-Hydroxysuccinimide-Biotin (NHS-Biotin) was acquired from Pierce (USA). Phospholipase D (PLD) was obtained from Calbiochem (Germany). Monoclonal antibody (mab) against EgAgB8/1 subunit (EB7 mab) was generously donated by Dr. Gualberto González (Cátedra de Inmunología, Facultad de Química, UdelaR). Flow cytometry antibodies were purchased from BD Biosciencies or Biolegend (USA). Phosphatidylcholine (PC) and phosphatidylserine (PS) were obtained from Avanti Polar Lipids (USA).

### Mice

Wild type C57BL/6J and BALB/c mice as well as LDL-receptor (LDLr) deficient mice on the C57BL/6J background (B6.129S7-Ldlrtm1Her) were acquired from Pasteur Institute of Montevideo (Uruguay). Animal manipulation and husbandry were done in accordance with the ethical committee guidelines of the Honorary Commission of Animal Experimentation (CHEA) from UdelaR.

### Parasite material

*E. granulosus* bovine hydatid cysts were obtained from liver and lungs of naturally infected animals, collected during the routine work of local abattoirs in Montevideo (Uruguay). Infertile HF were obtained by aseptic aspiration of the cyst content, preserved by addition of 5 mM EDTA and 20 μM BHT, and maintained at −20 °C until use.

### Purification of native EgAgB

Purification of native EgAgB was achieved employing a two-step procedure. A pool of individual HF (at least a volume of 2 L, collected from an average of 15 hydatid cysts) was prepared and clarified by centrifugation at 10000 g followed by filtration through 0.45 μm filter membrane (Millipore). The clarified HF was then fractionated by anion exchange chromatography on a Q-Sepharose column, using 20 mM phosphate, pH 7.5 containing 200 mM NaCl, 5 mM EDTA and 20 μM BHT as equilibration buffer, and changing the ionic strength to 500 mM NaCl in a single step for elution. The eluted fraction (f_QS_) was concentrated 10-fold using a Savant SpeedVac System and equilibrated in phosphate buffered saline (PBS) containing 5 mM EDTA and 20 μM BHT (PBS_EB_) with a PD-10 desalting column (Amersham, Biosciences). EgAgB was then purified by ultracentrifugation of f_QS_ in a KBr density gradient. 2.45 g of KBr were dissolved in 5 ml of f_QS_ in an ultracentrifuge tube and slowly covered with a solution containing 0.15 M NaCl and 0.42 M KBr. After ultracentrifugation (4 h at 332000 g) two yellowish bands were obtained. EgAgB was recovered in the yellow-brown, low density fraction. The homogeneity of EgAgB preparation was analysed by SDS-PAGE on 15 % polyacrylamide gels followed by Coomassie staining. At least two consecutive ultracentrifugation rounds were performed to achieve a good-quality EgAgB preparation (higher than 95 % purity). Finally, EgAgB was equilibrated in PBS_EB_ and maintained at 4 °C under N_2_ atmosphere until use. Five different EgAgB preparations were used during this work. The apolipoprotein composition of native EgAgB was determined by LC-MS/MS, using an LTQ-Orbitrap Velos mass spectrometer (Thermo Scientific). Proteins were identified using MaxQuant software (v.1.5.3.8) by searching MS and MS/MS data against the *Echinococcus* database; this database was built in house comprising all *Echinococcus granulosus* G1 sequences (published in www.genedb.org as EGU_proteins_29042013_products.fa) plus a total of 107 sequences, including polymorphic variations at the level of the mature products as well as the orthologous products in *E. granulosus* s.l. and *E. multilocularis* (available on NCBInr). Statistical analysis for protein identification was performed using Perseus (v. 1.4.0.11) on the basis of unique peptide MS intensities and the presence of a minimum of two unique peptides. For evaluating the abundance of EgAgB8 subunits in samples, intensity-based absolute quantification (iBAQ) was used since it has been reported as a useful label-free quantification method provided by MaxQuant [33]. The relative abundance of a particular EgAgB8 subunit was then estimated as the percentage of the total iBAQ intensity corresponding to EgAgB apolipoproteins.

For functional assays on macrophages native EgAgB was purified by immunoaffinity chromatography employing the EB7 mab as described by González et al. [13] (immunopurified EgAgB) to exclude pyrogens which may affect cell functions even at trace levels.

### Purification of recombinant lipid-free EgAgB8 subunits

Purification of recombinant lipid-free EgAgB8 subunits (rEgAgB8) was undertaken employing a protocol previously described [20]. Briefly, rEgAgB8/1, rEgAgB8/2 and rEgAgB8/3 subunits (corresponding to [Uniprot: Q9UA06, Q27275 and Q95NW6, respectively]) were purified as Glutathione-S-Transferase fusion proteins by affinity chromatography on immobilised glutathione and recovered by thrombin cleavage. Delipidation of rEgAgB8 subunits was achieved by reversed-phase high-performance liquid chromatography (RP-HPLC) in an HPLC System (Merck-Hitachi, Japan) with C8-bonded silica as stationary phase and water/acetonitrile/trifluoroacetic acid mobile phase. The purity of lipid-free rEgAgB8 subunits was monitored by SDS-PAGE on 15 % polyacrylamide gels followed by Coomassie staining. Lipid-free rEgAgB8 subunits were maintained at −80 °C until use.

### Biotin labelling of EgAgB

Biotinylation of native EgAgB was carried out by adding 80 μL of a fresh solution of NHS-Biotin (5 mg/mL in DMSO) per mg of protein in sodium carbonate 0.1 M, pH 9.0. In the case of lipid-free rEgAgB81, rEgAgB8/2 and rEgAgB8/3 subunits, we used an NHS-Biotin concentration sufficient to label a maximum of six lysines per molecule in each subunit. The mixture was incubated during 4 h at room temperature under continuous stirring. Excess NHS-Biotin was removed by extensive dialysis against PBS_EB_. Biotinylated rabbit IgG, as well as OVA labelled under the conditions described above were employed as controls. Protein biotinylation was monitored by adsorbing the biotinylated proteins on an ELISA microplate and using streptavidin-peroxidase and TMB for developing. Under these labelling conditions, the extent of biotinylation was similar between EgAgB (native or recombinant subunits) and the protein control (OVA), as wells as among rEgAgB8 subunits (Additional file 1).

### EgAgB treatment with phospholipase D

Native EgAgB was treated with PLD (10 U PLD/mg EgAgB) for 24 h at 37 °C in 30 mM Tris–HCl, 2 mM CaCl_2_, 100 mM NaCl, pH 8.0. After treatment, PLD was removed by ultracentrifugation of EgAgB particles in a KBr gradient as described above (EgAgB_PLD+_). In parallel EgAgB was treated without adding PLD (EgAgB_PLD-_) as a control of treatment. Effective PLD treatment was analysed by lipid extraction and high performance thin layer chromatography (HPTLC) as previously described [21].

### Purification of plasma lipoproteins

LDL and HDL were isolated from normal human plasma obtained from healthy volunteers after informed consent. Isolation was performed following a described procedure based on ultracentrifugation in a KBr density gradient [34]. After ultracentrifugation, LDL and HDL were extensively dialysed against PBS_EB_, maintained at 4 °C and under N_2_ atmosphere, and immediately used in competition binding assays as described below.

### Large unilamellar vesicles preparation

LUVs composed of PC (PC-LUVs) or PC and PS (50:50 mol:mol, PC/PS-LUVs) were prepared by extrusion through polycarbonate membranes of 100 nm pore diameter, employing mini extruder equipment (Avanti Polar Lipids), as previously described [35]. They were maintained at 4 °C under N_2_ atmosphere and immediately used in competition binding assays as described below.

### Isolation of mouse peritoneal cells

Isolation of mouse inflammatory cells was achieved following a procedure previously described [36, 37]. Briefly, C57BL/6J or B6.129S7-Ldlrtm1Her (LDLr^−/−^) mice were injected with 100 μL of Freund’s incomplete adjuvant to induce an appropriate recruitment of inflammatory cells into the peritoneal cavity. After 48 h mice were euthanized by cervical dislocation under anaesthesia and cells were obtained by peritoneal washes using PBS containing 2 mM EDTA and 2 % (v/v) FS. Mouse peritoneal cells were then immediately used in binding assays as described below.

### Cell culture

The human monocyte-like cell line THP-1 (American Type Culture Collection, USA) was maintained in RPMI 1640 culture medium containing 10 mM HEPES, 1.5 g/L sodium bicarbonate, 1 mM sodium pyruvate, 2 mM glutamine, penicillin/streptomycin/amphotericin B (100 U/mL, 0.1 mg/mL, 250 ng/mL, respectively) and 10 % (v/v) FS. Cells were maintained in a humidified 37 °C incubator with 5 % (v/v) CO_2_ and subcultured every 3–4 days to maintain density between 0.2–1.0 × 10^6^ cells/mL. For macrophage differentiation, cells were stimulated with PMA (50 ng/mL) for 72 h. Characterisation of PMA-differentiated macrophages was carried out by flow cytometry using specific antibodies for cell receptors in the conditions recommended by the manufacturer (BD Bioscience). THP-1 monocytes were CD14^low^CD32^high^CD64^high^ and they became CD14^−^CD64^med^CD32^med^ after PMA-differentiation as expected for macrophages. THP-1 monocytes and THP-1-derived macrophages were used in binding assays as described below.

### Binding of biotinylated proteins to cells

Binding assays were performed by flow cytometry using biotinylated proteins and mouse peritoneal cells, THP-1 monocytes or THP-1 derived macrophages. For THP-1 macrophages, cells were firstly detached using cold PBS containing 1 mM EDTA. All incubations and washing steps were carried out in binding buffer (BB, PBS containing 1 % (v/v) FS and 0.1 % NaN_3_). Cells were dispensed in 96-well conical bottom plates at 0.5, 0.75 or 1.0 × 10^6^ cells/well for THP-1 monocytes, macrophages and peritoneal cells respectively. Cells were incubated for 1 h at 4 °C with increasing concentrations of the biotinylated protein in duplicates or triplicates. After three washing steps with BB, protein binding was detected by incubation with an excess concentration of streptavidin-FITC for 45 min at 4 °C. In parallel, cells were incubated with biotinylated OVA for controlling unspecific binding. After washing, cells were examined using a FACSCalibur flow cytometer (BD Biosciences, USA) and data was analysed using Cell Quest software or the FlowJo™ package. In the case of peritoneal cells, cells were co-stained with anti-mouse F4/80 antibody conjugated to phycoerythrin (or its corresponding isotype control) which allow to analyse the binding to mouse peritoneal monocytes and macrophages by gating on F4/80^+^ cells. Lymphocytes were defined on the basis of their size (forward scatter), cell complexity (side scatter) and stain for F4/80 expression (negative cells). For comparison, the binding of biotinylated proteins was expressed as binding index, which corresponds to the ratio of the fluorescence intensity (geometric mean) of the sample relative to the control (cells incubated with BB).

### Binding assays using the EB7 monoclonal antibody

Similar binding assays were performed using THP-1 monocytes and macrophages, and the EB7 mab for developing. Briefly, cells were incubated with unlabelled native EgAgB, EgAgB_PLD+_, EgAgB_PLD-_ or lipid-free rEgAgB8/1 subunit for 1 h at 4 °C in duplicate or triplicate. Protein binding was detected by incubation with EB7 mab for 45 min at 4 °C, followed by incubation with goat anti-mouse IgG/IgM conjugated to FITC (1/50) for 45 min at 4 °C. In parallel, controls were carried out by adding BB instead of EgAgB, or mouse IgG1 kappa isotype control instead of EB7 mab. After washing, cells were analysed by flow cytometry as stated above. The binding of EgAgB to the cells was expressed as binding index, as previously described.

### Competition binding assays

Competition binding assays were performed by co-incubating biotinylated and unlabelled EgAgB to examine binding specificity. All incubations were performed at 4 °C and using BB. For these experiments, we used THP-1 monocytes or macrophages, the minimum concentration of biotinylated EgAgB needed for saturation, and a ratio of unlabelled:biotinylated EgAgB ranging from 0.5:1 to 4:1. Co-incubation using unlabelled OVA and biotinylated EgAgB (in a mass ratio of 4:1, which corresponds to about 20-fold molar excess) was undertaken as a control. For comparison similar competition assays were carried out with unlabelled and biotinylated OVA. In addition, competition binding assays were performed using LUVs, plasma lipoproteins (HDL and LDL) and ligands of lipoprotein receptors (lactoferrin and polyinosinic acid) in an excess concentration as indicated. In the latter competition assays cells were not co-incubated with EgAgB and the competitor to prevent possible interactions between EgAgB particles and LUVs or lipoproteins; instead, after a pre-incubation with the competitor (30 min), cells were centrifuged and subsequently re-suspended in BB containing the biotinylated EgAgB. All assays were carried out in duplicate or triplicate.

### Cytokine secretion assays

For cytokine secretion assays, THP-1 monocytes (0.5 × 10^6^ cells/well) were differentiated with PMA for 72 h and then cultured in medium without PMA for 24 h. The resultant macrophages were stimulated with PMA (50 ng/mL) or LPS (0.1 μg/mL) in the absence or presence of increasing concentrations of immunopurified EgAgB (1, 10 or 20 μg/mL) for 12 h at 37 °C with 5 % (v/v) CO_2_. In parallel cells were incubated with PBS_EB_ or immunopurified EgAgB (20 μg/mL) as controls. The levels of IL-1β, TNFα and IL-10 in culture supernatants were determined by capture ELISA employing OptEIA kits (BD Biosciences), according to the manufacturer’s instructions.

### Arginase activity assay

Arginase activity was evaluated in macrophage cultures employing the method described by Corraliza and collaborators [38]. Briefly, bone marrow-derived macrophages (BMDM) were generated by differentiation of bone marrow precursors from 8 to 10 week old BALB/c mice for 7 days in the presence of conditioned medium of the M-CSF-secreting L929 cell line. BMDM were cultured in the presence of immunopurified EgAgB (0.2–20 μg/mL), murine IL-4 (2.5 ng/ml, as a control of alternative activation) or PBS_EB_, and after 24 h they were lysed with 0.1 % Triton X-100. Cells were scraped into 10 mM MnCl_2_, 50 mM Tris–HCl, pH 7.5 and heated to 56 °C for 10 min to activate arginase-I. Substrate hydrolysis was performed by adding 0.5 M L-arginine, pH 9.7 to the cell lysate followed by 60 min incubation at 37 °C. The reaction was stopped by adding an acid mixture containing H_2_SO_4_, H_3_PO_4_ and H_2_O (1:3:7). After adding α-isonitrosopropriophenone, samples were heated to 110 °C for 30 min, and urea content was then measured spectrophotometrically at 540 nm. Enzymatic activity (U) was determined as μmoles of urea generated per minute, employing a urea calibration curve. Arginase activity was then expressed as mU per million of BMDM. In parallel, nitrite levels in BMDM culture supernatants were determined employing Griess assay according to Green and collaborators [39].

### Statistical analysis

For all studies, data were obtained from at least three independent experiments and expressed as means ± standard error of the mean (SEM). Most statistical analyses were undertaken employing Graph Prism software. In most studies one-way analysis of variance (ANOVA) followed by the indicated post hoc test (Tukey’s or Dunnett’s test) was used to evaluate the binding ability of EgAgB (native, the recombinant subunits or PLD-treated EgAgB) to cells. Two-way ANOVA followed by Bonferoni post hoc test was used for comparing the binding index exhibited by EgAgB at different doses. For competition assays one way ANOVA and restricted maximum likelihood (REML) test were used, employing JMP software. Significance was defined as *p* < 0.05 and was indicated in each figure.

## Results and discussion

Infertile bovine cysts constitute the main parasite material to which we have access in our country. Despite EgAgB being the most abundant parasite component of HF, we only recovered between 1.5 and 2.5 mg of EgAgB per L of infertile HF. Studies described in this work required preparation of five independent batches of native EgAgB, each batch from a representative number of individual cysts (at least 10 cysts, 15 cysts on average). Due to the high diversity of EgAgB family, we firstly characterised the protein moiety of native EgAgB, in order to know which EgAgB8 apolipoproteins are relevant for binding studies. Characterisation by LC-MS/MS analysis of these batches demonstrated the presence of EgAgB8/1 to EgAgB8/5 (Table 1), where EgAgB8/1 was the predominant subunit according to the iBAQ parameter, reaching a relative abundance of 96 %. Interestingly, we found similar results when the protein composition of native EgAgB present in fertile HF was analysed by LC-MS/MS; although all EgAgB8 subunits were present, EgAgB8/1 was over-represented (Ana Maite Folle, unpublished observations). Taken together, these results support that EgAgB8/1 constitutes the bulk of the apolipoprotein component found in EgAgB derived from HF and it is mainly synthesised by the GL.

**Table 1:** Identification of EgAgB8 apolipoproteins in Infertile-f_QS_ by LC-MS/MS

Apolipoprotein	Uniprot Accession Number	Molecular Mass (Da)	pI	Intensity (% coverage)	iBAQ	Unique peptides
EgAgB8/1	Q5EKQ4	7589.9	8.31	29.7 (53.5)	28.1	DDGLTSTSR, DPLGQKVVDLLK, DPLGQKVVDLLKELEEVFQLLR, ELEEVFQLLR, ELEEVFQLLRK, VVDLLKELEEVFQLLR, VVDLLKELEEVFQLLRK, YFFERDPLGQK
	Q9UA06^a^	7555.9	8.31			
EgAgB8/2	Q5EKP1	7906.2	9.42	21.1 (37.7)	19.5	AHMGQVVK, AHMGQVVKKDFFRNDPLGQR, LVALGNDLTAICQK, NDPLGQR, NLVEEKDDDSK, YVKNLVEEK, YVKNLVEEKDDDSK
	Q27275^b^	8193.5	9.37			
EgAgB8/3	Q95NW6^c^	7858.2	8.02	24.0 (33.9)	22.4	DDDDDEVTK, DVASVCEMVR, HFFQSDPLGK, HFFQSDPLGKK
	A0A068X006–1	6712.8	6.78			
EgAgB8/4	Q6Q0H5	8353.66	6.20	24.1 (27.3)	22.6	DFFRSDPLGQR, DLLEEEEEEDDSK, DLTAICQK, YVKDLLEEEEEEDDSK
EgAgB8/5	Q5EKP9	7657.07	9.56	21.0 (14.7)	19.4	EVASVCQMVR

The following proteins (Uniprot accession numbers) generate identical mature products to that indicated with a superscript letter: a U6JQF4 and Q5S577, b Q5EKN4, C1KBK4, Q6Q0H3 and Q6Q0I3, c Q5EKQ8, Q5EKR1, Q5EKR3 and Q95W92

Molecular mass and isoelectric point (pI) of mature proteins were calculated using the “compute pI/MW” Expasy tool (http://web.expasy.org/compute_pi/)

Intensity values correspond to log2 of summed XIC (extracted ion current) of all isotope clusters associated to the corresponded protein and are given as median of quintuplicates. The iBAQ values were also log2-transformed and their relative standard deviation was ≤ 1.1 %

Coverage values correspond to the percentage of the protein sequence that is covered by the identified peptides

### Native EgAgB binds to F4/80^+^ mouse peritoneal cells

**Fig. 1:**
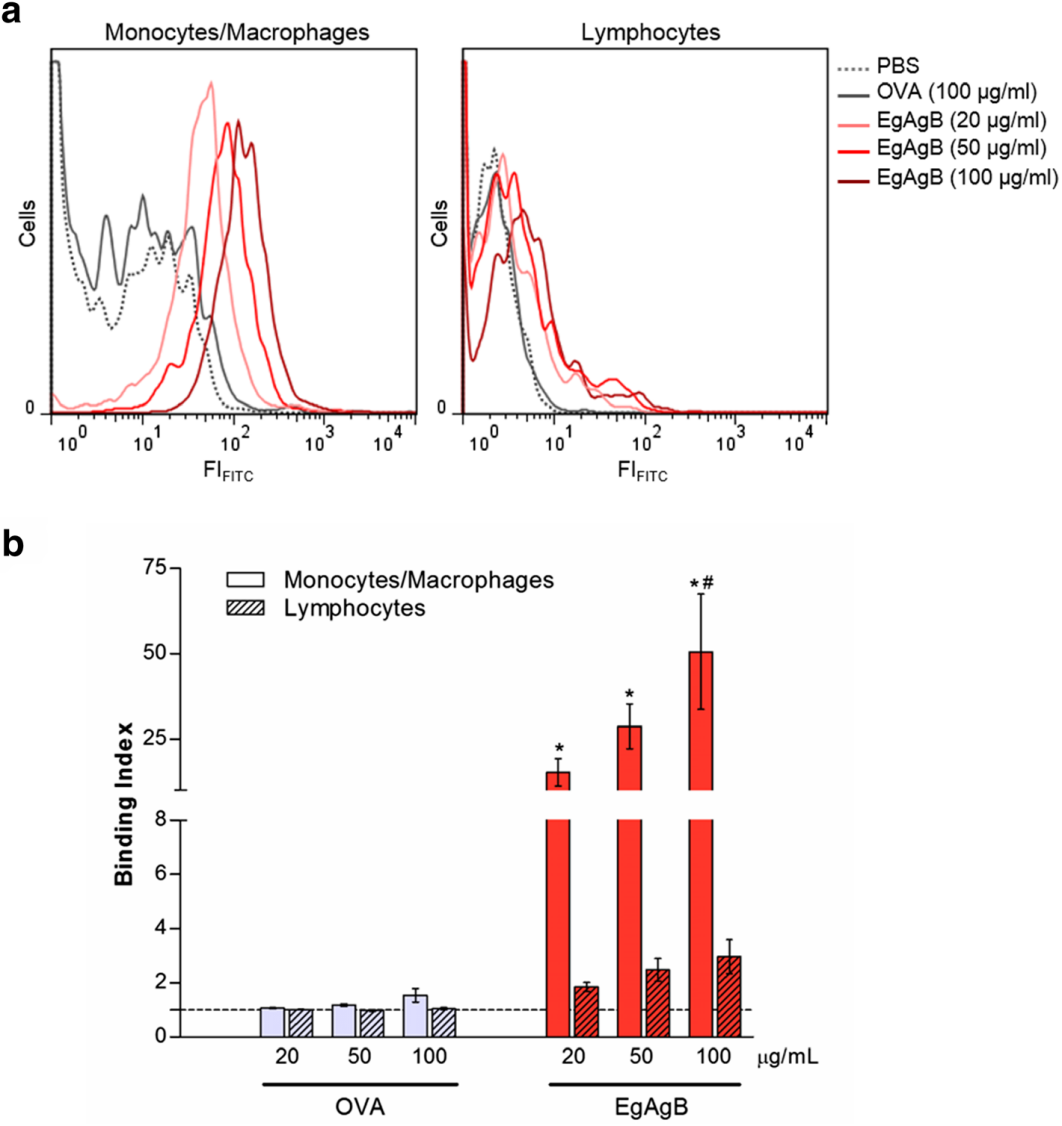
Binding of native EgAgB to mouse inflammatory cells. Binding of native EgAgB to mouse inflammatory cells was evaluated using peritoneal inflammatory cells and biotinylated EgAgB (20, 50 and 100 μg/mL). Biotinylated OVA was used as a control. Protein binding was detected by incubation with an excess concentration of streptavidin-FITC. Macrophages and monocytes were selected by co-staining with anti-F4/80 antibody conjugated to phycoerythrin. Lymphocytes were identified on the basis of their size (FSC), complexity (SSC) and negative stain for F4/80. **a** Histograms with the distribution of cell population as function of FITC fluorescence for controls (grey) and EgAgB-treated cells (red). Histograms are representative of three independent experiments for monocytes/macrophages (F4/80^+^) and lymphocytes (F4/80^−^). **b** EgAgB binding to monocytes/macrophages or lymphocytes are shown as binding index (increment of the fluorescence relative to the control with BB), corresponding to the mean values ± SEM of three independent experiments. Binding of OVA (grey) and native EgAgB (red) is shown for monocytes/macrophages (empty bars) and for lymphocytes (filled bars). Asterisks (*) denote significant differences with respect to the control (one way ANOVA followed by Dunnett’s post-test, *p* < 0.05), while number signs (#) denotes significant differences when comparing the binding to monocytes/macrophage to that to lymphocytes (one-way ANOVA analysis, followed by Tukey’s post-test (*p* < 0.05)

As noted above, EgAgB function may be linked to the parasite adaptation response to host environment, by mediating lipid mobilisation from host to parasite tissues [21, 23] and/or by contributing to modulate the effector mechanisms displayed by immune cells [29–32]. Because evidence of EgAgB effects on myeloid cells exists, we initially attempted to examine EgAgB interactions with inflammatory cells. For that purpose, we performed fluorescence binding assays employing mouse peritoneal cells, recovered after adjuvant-induced acute inflammation, biotinylated EgAgB and streptavidin-FITC for development. Employing biotinylated EgAgB offered the advantage to examine the binding of EgAgB independently of the apolipoprotein composition of individual particles, since all EgAgB8 isoforms contain various lysine residues [22] to allow easy protein biotinylation. All incubations were performed at 4 °C in the presence of NaN_3_ to avoid EgAgB endocytosis. We used the binding index, defined as the increment of the fluorescence relative to the control, in order to compare the binding activity of EgAgB and OVA. We found that EgAgB binding index to inflammatory F4/80^+^ cells (including mainly macrophages, but also inflammatory monocytes) was more than 20-fold higher than that observed for OVA used as control, and this difference was statistically significant (Fig. 1). Furthermore, EgAgB binding to inflammatory F4/80^+^ cells was significantly higher than that to inflammatory lymphocytes. Altogether, these results indicated that monocytes and macrophages recruited in an inflammatory site are able to interact with native EgAgB (Fig. 1).

### Native EgAgB binds to THP-1 monocytes and macrophages

**Fig. 2:**
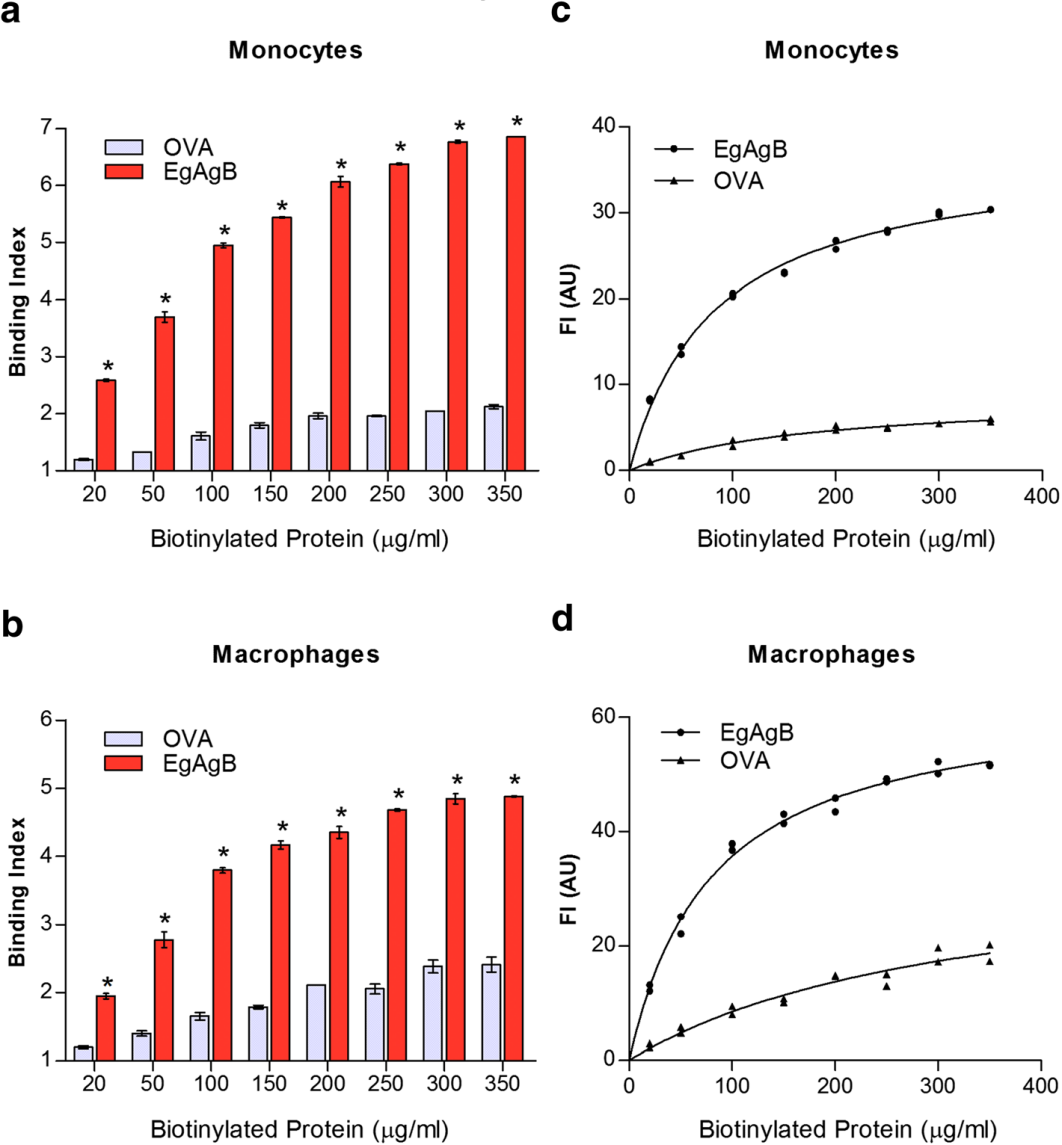
Binding of native EgAgB to THP-1 derived monocytes and macrophages. Binding of native EgAgB to THP-1 monocytes and macrophages was evaluated using biotinylated EgAgB or biotinylated OVA as control (20–350 μg/ml). In (**a**) and (**b**) Binding indexes of OVA (grey) or native EgAgB (red) for monocytes and macrophages, respectively; data are expressed as mean values ± SEM of triplicates. Asterisks (*) denote significant differences in binding indexes of native EgAgB with respect to the control (two-way ANOVA followed by Bonferroni’s post-test, *p* < 0.05). (**c**), (**d**) Titration curves obtained for OVA (▲) or EgAgB (●) binding to monocytes or macrophages, respectively. Several fitting models of Graph Prism software were tested and the “binding saturation with one side-specific binding” model showed the best R^2^ (0.992). The solid line corresponds to the theoretical binding curve obtained for each protein. One representative experiment of three is shown in each panel

Since *E. granulosus* hydatid cyst is extremely well adapted to host inflammation [40], resident immune cells in the cyst vicinity may not be exposed to a strong pro-inflammatory environment once the parasite has been established. Thus we studied the EgAgB binding activity to non-activated macrophages and monocytes using similar fluorescence binding assays and the monocyte-like THP-1 cell line of human origin. When comparing the binding index at the same protein dose, and in a wide range of ligand concentrations (20 – 350 μg/ml), we found that biotinylated EgAgB showed a higher binding index to THP-1 monocytes and macrophages than biotinylated OVA (Fig. 2a and b, respectively). In addition, when the fluorescence intensity was plotted vs. EgAgB concentration saturation curves were obtained (Fig. 2c and d), suggesting that EgAgB binding to monocytes and macrophages involved specific interactions. The apparent dissociation constant (K_d_) determined from both curves was similar (83 ± 5 and 79 ± 5 μg/ml for monocytes and macrophages, respectively), which corresponds to ~3 ×10^−7^ M, considering an average molecular mass of 230 kDa for the native lipoprotein [21]. In comparison, in the conditions of our assay OVA showed a lower affinity for THP-1 cells (with a K_d_ of 175 ± 25 and 327 ± 92 μg/ml for monocytes and macrophages, respectively), corresponding to ~10^−6^ M, an order of magnitude lower than for EgAgB. Taken together, these results showed that EgAgB binds specifically to THP-1 monocytes and macrophages, with a K_d_ ~10^−7^ M. This value likely represents an average of the affinities of EgAgB, since the native lipoprotein comprises a heterogeneous mixture of particles, with molecular masses between 400 and 200 kDa [21]. As an alternative to using biotinylated ligand, EgAgB binding to monocytes and macrophages was examined by developing the interaction with an anti-EgAgB monoclonal antibody (EB7 mab), which recognizes EgAgB8/1 -the predominant subunit found in the native antigen-, but not EgAgB8/2 [13] or EgAgB8/3 (Additional file 2). No data is available in relation to the recognition of EB7 mab for EgAgB8/4, but it is unlikely since EgAgB8/4 is more similar to EgAgB8/2 than to EgAgB8/1 [18]. Using this approach, we found that the binding index was significantly higher for native EgAgB than for the control (mouse IgG) and increased in a dose-dependent manner (Additional file 3). Overall, results show that monocytes and macrophages are able to bind native EgAgB, in particular those lipoprotein particles containing EgAgB8/1.

**Fig. 3:**
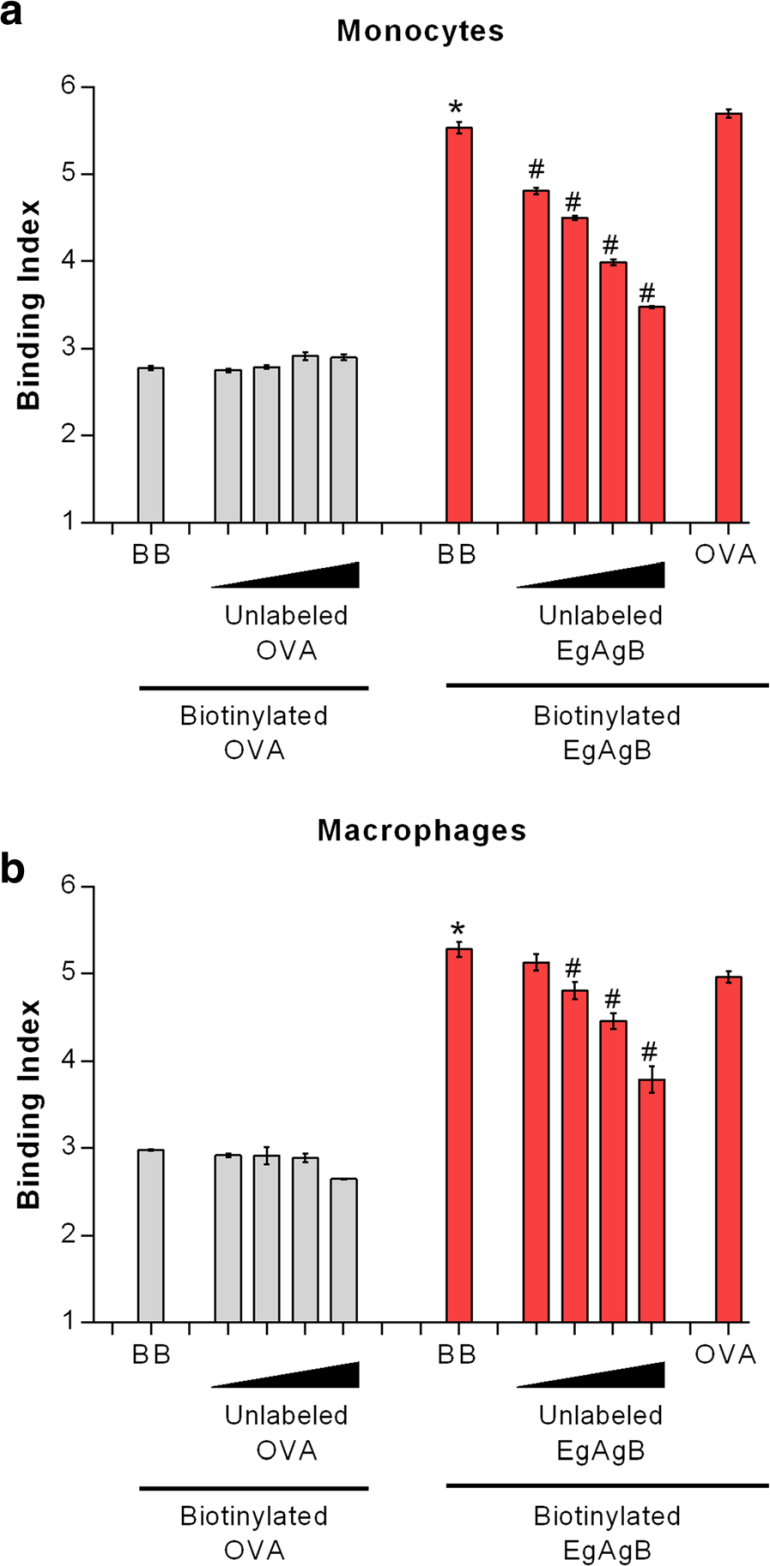
Competition binding assays for studying binding specificity. In these assays the binding of biotinylated EgAgB (in a fixed concentration 250 μg/ml) was competed out by the unlabelled lipoprotein (at concentrations between 125 and 1000 μg/ml) or OVA (1000 μg/ml) as a control (red bars). In parallel, similar competition assays were carried out using biotinylated OVA (grey bars). **a** Assays for THP-1 monocytes. **b** Assays for THP-1 macrophages. Results are expressed as binding index, and correspond to mean values ± SEM of triplicates. Asterisks (*) indicate significant differences with respect to the binding of biotinylated OVA used as a control, while number signs (#) indicate significant differences with respect to biotinylated EgAgB incubated in the absence of the unlabelled lipoprotein (one-way ANOVA followed by Tukey’s post-test, *p* < 0.05). One representative experiment of two is shown in each panel

In order to confirm a specific interaction of monocytes and macrophages with EgAgB, we performed a competition assay in which cells were co-incubated with biotinylated and unlabelled EgAgB. For this assay we used the minimum concentration of biotinylated EgAgB needed to achieve saturation and employed a ratio ranging from 0.5:1 to 4:1 of unlabelled:biotinylated protein. In parallel, cells were co-incubated with biotinylated EgAgB and OVA as a control. We found that unlabelled EgAgB was able to compete with biotinylated EgAgB for the binding of monocytes and macrophages (Fig. 3a and b, respectively). In contrast, the binding of biotinylated OVA was not inhibited by unlabelled OVA. Moreover, no inhibition of EgAgB binding to monocytes and macrophages was observed when an excess of OVA was used for competition (Fig. 3a and b). Overall, these results indicated that THP-1 monocytes and macrophages are able to specifically bind native EgAgB, suggesting that these cells possess at least one surface receptor for EgAgB.

#### EgAgB apolipoproteins directly contribute to EgAgB binding to cells

Native EgAgB is a complex lipoprotein particle composed of around a dozen EgAgB8 subunits, which are likely exposed at the outer hydrophilic surface of the lipoprotein [21]. The involvement of these subunits in EgAgB binding to monocytes and macrophages was examined using biotinylated lipid-free rEgAgB8/1, rEgAgB8/2 and rEgAgB8/3 subunits. For that purpose, rEgAgB8 subunits were labelled with biotin in conditions to generate a maximum of six biotinylated lysines residues per molecule, attempting to avoid a significant perturbation of the protein structure. Since the yield of rEgAgB8/1 expression and delipidation procedures was much lower in comparison with that obtained for rEgAgB8/2 and rEgAgB8/3, some assays were carried out only with the latter subunits. It is relevant here that from the amino acid sequence, EgAgB8/3 is more similar to EgAgB8/1 than to EgAgB8/2 [18].

**Fig. 4:**
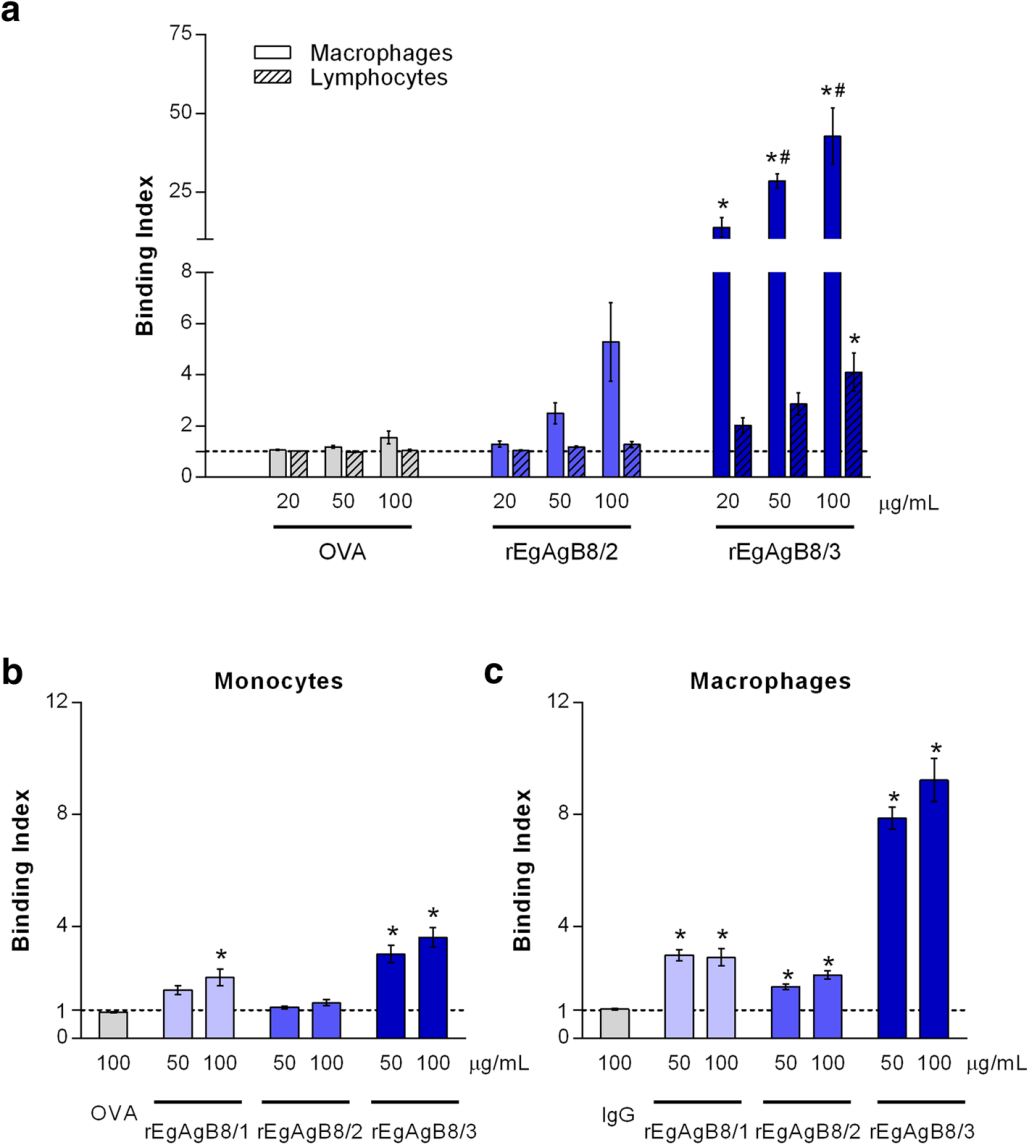
Analysis of the ability of monocytes and macrophages to bind rEgAgB8 subunits. Binding assays were performed using biotinylated lipid-free rEgAgB8/1, rEgAgB8/2 and rEgAgB8/3. **a** Binding index of rEgAgB8 subunits (20, 50 and 100 μg/ml) to mouse peritoneal inflammatory cells is shown. Data are expressed as mean ± SEM of three independent experiments. Asterisks (*) indicate significant differences with respect to the control (OVA) according to the analysis by one-way ANOVA followed by Dunnett’s post-test (*p* < 0.05). Number signs (#) denotes significant differences between binding to monocytes/macrophage and binding to lymphocytes, according to one-way ANOVA analysis, followed by Tukey’s post-test (*p* < 0.05). (**b**), (**c**) Binding indexes of rEgAgB8 subunits to THP-1 derived monocytes and macrophages, respectively. Data are expressed as mean ± SEM of three independent experiments. Asterisks (*) indicate significant differences with respect to the control (OVA) (one-way ANOVA followed by Dunnett’s post-test, *p* < 0.05)

When analysing the binding of rEgAgB8 subunits to peritoneal F4/80^+^ macrophages we found that rEgAgB8/3 behaved similarly to native EgAgB; rEgAgB8/3 binding index increased in a dose-dependent manner, being significantly higher than that for OVA (Fig. 4a). Moreover, rEgAgB8/3 binding to macrophages was significantly higher than to lymphocytes (Fig. 4a). On the other hand, binding of rEgAgB8/2 to macrophages was not observed; the binding index showed a trend to increase in a dose dependent manner, but this trend did not reach statistical significance (Fig. 4a). The different behaviour between rEgAgB8/2 and rEgAgB8/3 was also found when we used THP-1 cells for binding studies (Fig. 4b and c). rEgAgB8/3, but also rEgAgB8/1 were capable of binding to both monocytes and macrophages in a dose dependent manner. In contrast, rEgAgB8/2 did not bind to monocytes and showed a slight binding to macrophages. Taken into account that the extent of biotinylation of all subunits was similar (even a bit lower for rEgAgB8/3, Additional file 1), these results show that rEgAgB8/2 has a lower ability to interact with monocytes and macrophages than rEgAgB8/1 and EgAgB8/3, supporting the existence of differences in the biological properties of EgAgB subfamilies. However, it cannot be discarded that biotinylation would affect the binding properties of rEgAgB8/2 in a greater extent than those of EgAgB8/1 and EgAgB8/3. The capacity of rEgAgB8/1 to bind to THP-1 monocytes and macrophages was confirmed using EB7 mab to analyse the interaction (Additional file 4). The observed similarities and differences in cell interaction among EgAgB subunits are in accordance with the degree of identity between members of EgAgB family; as stated above EgAgB8/1 and EgAgB8/3 are more similar to each other than to EgAgB8/2. Taking into account that EgAgB8/1 was the predominant apolipoprotein found in the native EgAgB present in the HF, our results indicate that monocytes and macrophages may be mainly capable of recognising native EgAgB through EgAgB8/1. EgAgB8/3 might contribute to this recognition as well; although this subunit is poorly represented in the native EgAgB purified from HF, we cannot rule out that the composition of EgAgB released from the cyst towards host tissues may vary at distinct time points during infection or between different intermediate hosts.

### EgAgB phospholipids provide an adequate environment for EgAgB binding to cells

**Fig. 5:**
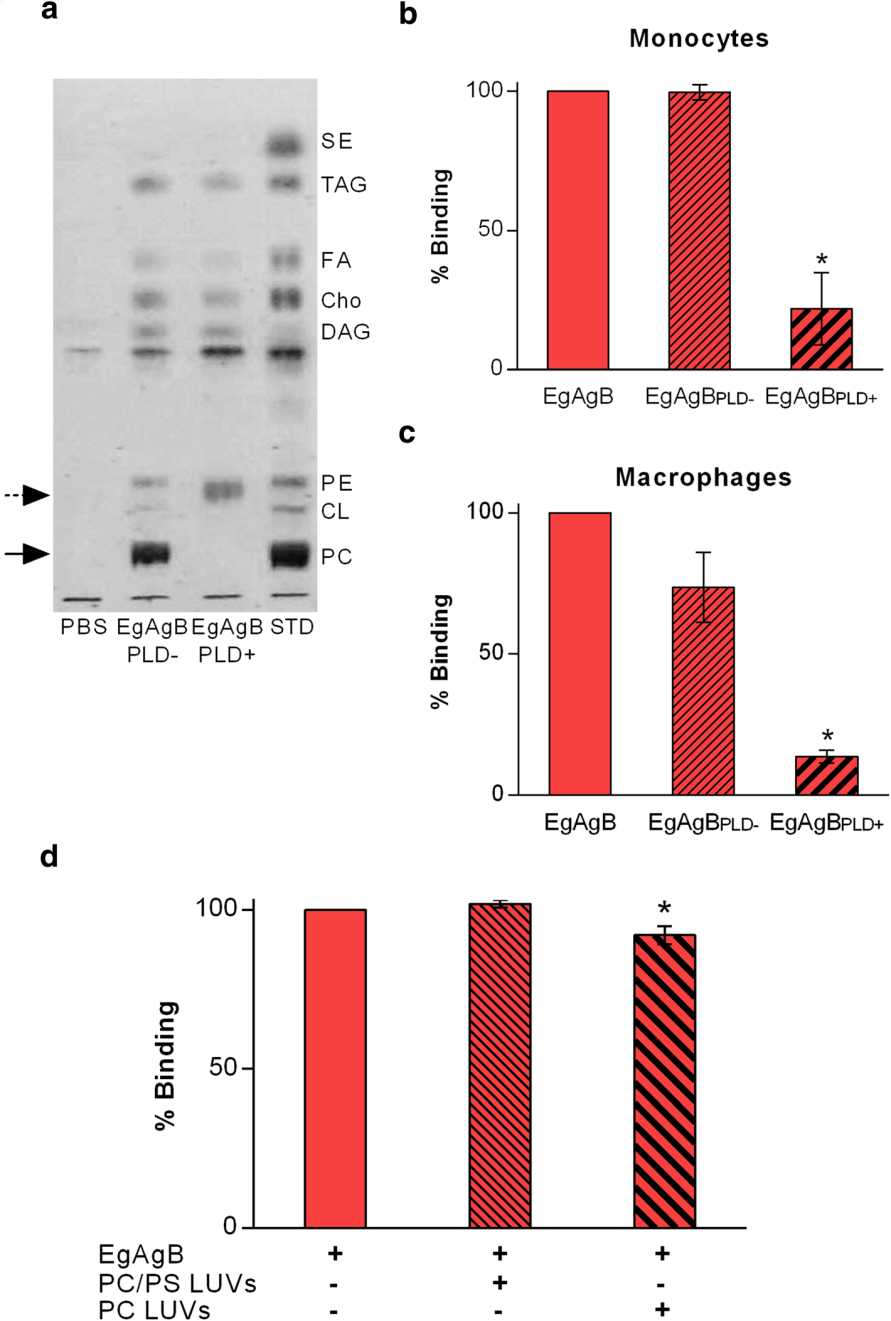
Contribution of phospholipid moiety in EgAgB binding to monocytes and macrophages. EgAgB was treated with PLD to remove the polar head group from phospholipids. **a** HPTLC analysis of the lipid fractions extracted from EgAgB_PLD-_ and EgAgB_PLD+_. Lipid bands were visualised using iodine vapour and identified by comparison with the standards (STD). The solid arrow indicates the band corresponding to phosphatidylcholine, whereas the dotted arrow indicates the band corresponding to phosphatidic acid. (**b**), (**c**) Binding of EgAgB_PLD-_ and EgAgB_PLD+_ to monocytes and macrophages, respectively. Binding was detected employing EB7 mab followed by incubation with anti-IgG/IgM-FITC. Results are expressed as percentage of binding (% binding), where 100 % corresponds to the binding of native EgAgB without any treatment. Data are expressed as mean ± SEM of three independent experiments. Asterisks (*) indicate significant differences with respect to the control (EgAgB_PLD-_) according to one-way ANOVA analysis followed by Dunnett’s post-test (*p* < 0.05). **d** Competition binding assays employing PC-LUVs and PC/PS-LUVs. Results are expressed as percentage of binding (% binding), where 100 % corresponds to the binding of native EgAgB in the absence of LUVs. Data are expressed as mean ± SEM of three independent experiments. Asterisks (*) indicate significant differences with respect to the 100 % of binding (without LUVs.) in accordance with one-way ANOVA analysis followed by Dunnett’s post-test (*p* < 0.05). Abbreviations: Cho (cholesterol); FA (free fatty acids); DAG (diacylglycerols); SE (sterol esters); TAG (triacylglycerols); PC (phosphatidylcholine); CL (cardiolipin) and PE (phsophatidylethanolamine)

In accordance with the structural organisation proposed for native EgAgB, phospholipids may form the outer layer of the lipoprotein particle in which EgAgB subunits are embedded. Therefore, phospholipids may play a relevant role in EgAgB binding to target cells by contributing to the exposure of apolipoprotein domains towards the hydrophilic milieu or by making direct contacts with cell receptors. To analyse the involvement of EgAgB phospholipids in the binding of the native lipoprotein to monocytes and macrophages, we treated EgAgB with PLD to remove the polar head group from phospholipids, yielding phosphatidic acid. PLD-treated EgAgB was then repurified by ultracentrifugation in a KBr gradient to ensure the recovery of the whole lipoprotein particle after treatment. Analysis by HPTLC of the lipid moiety of EgAgB_PLD+_ showed that PLD-treatment worked efficiently since it caused a strong alteration in the phospholipid composition of EgAgB. All phospholipid classes present in the mock control (EgAgB_PLD-_) disappeared after PLD treatment being substituted by a unique component that exhibited a migration pattern compatible with phosphatidic acid in the assayed chromatographic conditions (Fig. 5a, dotted black arrow) [41]. The analysis of EgAgB_PLD-_ and EgAgB_PLD+_ binding to THP-1 monocytes and macrophages revealed significant differences between these lipoprotein particles. EgAgB_PLD-_ bound to monocytes and macrophages to a similar extent to native EgAgB, whereas EgAgB_PLD+_ lost around 80 % of the binding capacity (Fig. 5b and c). It cannot be excluded that this detrimental effect caused by PLD treatment was a consequence of the negative charge generated by the formation of phosphatidic acid. Therefore, our results suggest that phospholipids may be directly involved in EgAgB-cell interactions or they may contribute indirectly, by generating an adequate framework for the recognition of EgAgB8 subunits in the surface of the lipoprotein particle. In order to evaluate whether phospholipids participate directly in EgAgB binding to cells, we performed competition assays employing PC-LUVs, which may model the PC enriched-phospholipid layer exposed on EgAgB, and PC/PS-LUVs for comparison. We found that pre-incubation of monocytes with PC/PS-LUVs, using a mass ratio of 5:1 LUVs:EgAgB, did not alter EgAgB binding to cells. PC-LUVs however caused a slight reduction (about 10 %) in EgAgB binding (Fig. 5d), which was not comparable to that caused by PLD treatment. These results suggest that EgAgB binding to monocytes may not be dependent on cell interactions with phospholipids exposed at the surface of EgAgB particle. On the other hand, the fact that PC-LUVs, but not PC/PS-LUVs caused a modest, but significant inhibition on EgAgB binding, suggests that electrostatic forces may play a role in EgAgB-cell interactions.

In summary, in accordance with the results described above, monocytes and macrophages are able to recognise specifically native EgAgB, through motifs belonging to EgAgB8 apolipoproteins rather than to the lipid components. In particular, the binding activity was found in EgAgB8/1, which is the most abundant apolipoprotein of the native EgAgB present in HF. Electrostatic interactions between EgAgB8 subunits and phospholipids may contribute to an adequate assembly and/or exposure of EgAgB subunits in the particle surface, which agrees with the predicted formation of negative and positive charge regions in all apolipoproteins by *in silico* structure modelling [23]. On the other hand, the fact that the binding activity motifs seem to be present in rEgAgB8 apolipoproteins suggests that the intact lipoprotein structure is not essential for cell binding. This might explain why denatured EgAgB preparations showed modulatory effects on immune cells [30, 31]. In any case, these protein motifs seem to be resistant to high temperature, SDS and electroelution. Nevertheless, the biological effects triggered by these motifs on immune cells may be partially different to that caused by the native EgAgB, as the latter might bind to a different set of cell receptors, imprinting different signals into the cell.

### Lipoprotein receptors are likely involved in EgAgB binding to cells

**Fig. 6:**
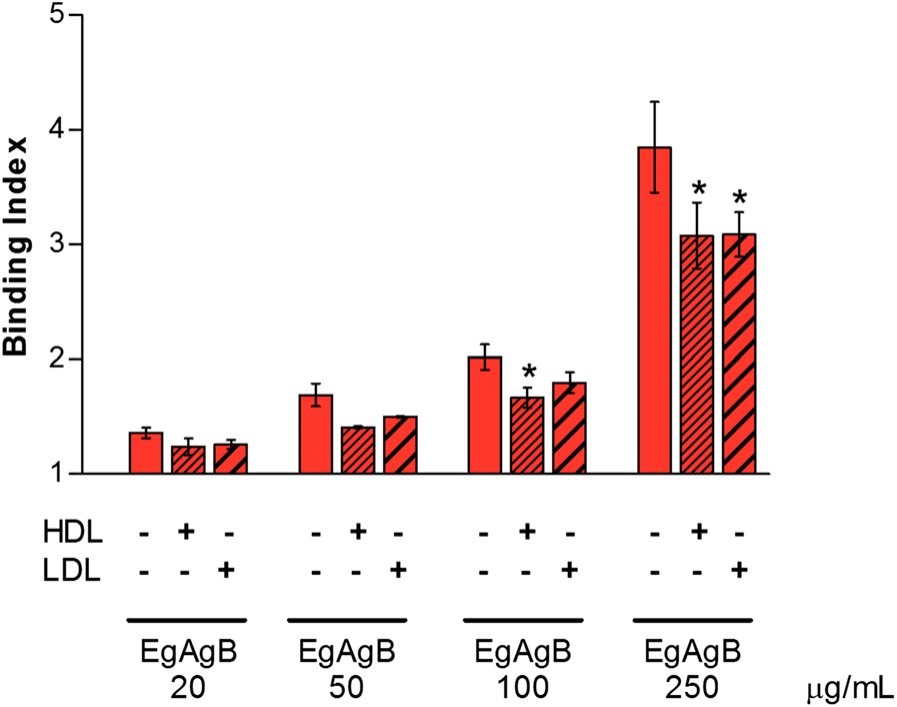
Participation of plasma lipoprotein receptors in EgAgB binding to monocytes. THP-1 monocytes were pre-incubated with HDL and LDL (500 μg/ml), centrifuged and then incubated with biotinylated EgAgB (20, 50 100 and 250 μg/ml). Results are expressed as binding indexes and correspond to mean values ± SEM of three independent experiments. Asterisks (*) indicate significant differences with respect to the control incubated without lipoproteins, according to analysis by REML test employing JMP software

Since EgAgB lipoprotein share physicochemical properties with plasma lipoproteins, particularly with HDL particles [21], we performed competition experiments with HDL and LDL in order to assess whether lipoprotein receptors are involved in EgAgB-cell interactions. For these experiments, THP-1 monocytes were pre-incubated with HDL and LDL, centrifuged and then incubated with biotinylated EgAgB. We did not undertake a co-incubation of EgAgB with HDL or LDL to avoid putative EgAgB-lipoprotein interactions that may reduce EgAgB binding without the involvement of lipoprotein receptors. We found that both HDL and LDL partially inhibited binding of EgAgB to monocytes; they caused a modest, but statistically significant inhibition (Fig. 6). These results suggest that EgAgB binding to monocytes and macrophages is at least partially mediated by receptors shared with LDL and HDL.

Although EgAgB8 apolipoproteins do not have similarity at the amino acid sequence level with HDL and/or LDL apolipoprotein components, they exhibit some biochemical features similar to exchangeable plasma apolipoproteins, which are components of both HDL and LDL [42, 43]. Indeed, exchangeable apolipoproteins include small, α-helix rich proteins (some of them less than 10 kDa in mass) [42], and, lipid-free EgAgB apolipoproteins have substantial amounts (about 40 %) of α-helical structure [20], which increases when bound to lipids (about 65 % for the native lipoprotein) [44]. Furthermore, *in silico* analysis of the secondary structure of EgAgB8/1, EgAgB8/2 and EgAgB8/3 [22, 44] suggests that these apolipoproteins would adopt amphipathic α-helical structures, a feature associated with the exchangeability of apolipoproteins such as Apo AI and Apo E (reviewed by Jonas and Phillips, [42]). Similarities in the distribution of polar and/or charged amino acids between EgAgB8 and exchangeable apolipoproteins present in HDL and LDL might explain why these evolutionarily distant lipoproteins share receptors in monocyte and macrophages. On the other hand, there is a wide range of receptors in innate immune cells involved in lipoprotein recognition, including families exhibiting a relatively promiscuous ligand specificity, such as the LDL-receptor family and scavenger receptors [45, 46]. Since EgAgB8 subunits seem to adopt tertiary structures in which positive and negative charges are grouped [23], we explored the involvement of lipoprotein receptors containing domains with affinity for positively-charged (LDL receptor related protein 1 [LRP1] and LDLr [45]) or negatively-charged (class A scavenger receptor [SR-A] [45, 46]) motifs. For that purpose we carried out competitive binding assays using an excess of lactoferrin and polyinosinic acid, which are high-affinity ligands for LRP-1 and SR-A respectively. As shown in Additional file 5, we found no evidence of the involvement of any of these receptors. Furthermore, using monocytes and macrophages from LDLr-deficient mice, we explored the involvement of LDLr in the binding of native EgAgB. No evidence was found as inflammatory peritoneal F4/80^+^ cells recovered from LDLr-deficient mice exhibited similar EgAgB binding activity to those from controls (also shown in Additional file 5). Further studies for identifying EgAgB receptor(s) are needed. This identification together with the characterisation of the signalling pathway triggered by EgAgB recognition are of foremost importance to elucidate the role played by EgAgB in parasite biology.

### Exposure to native EgAgB affects the functional properties of macrophages

**Fig. 7:**
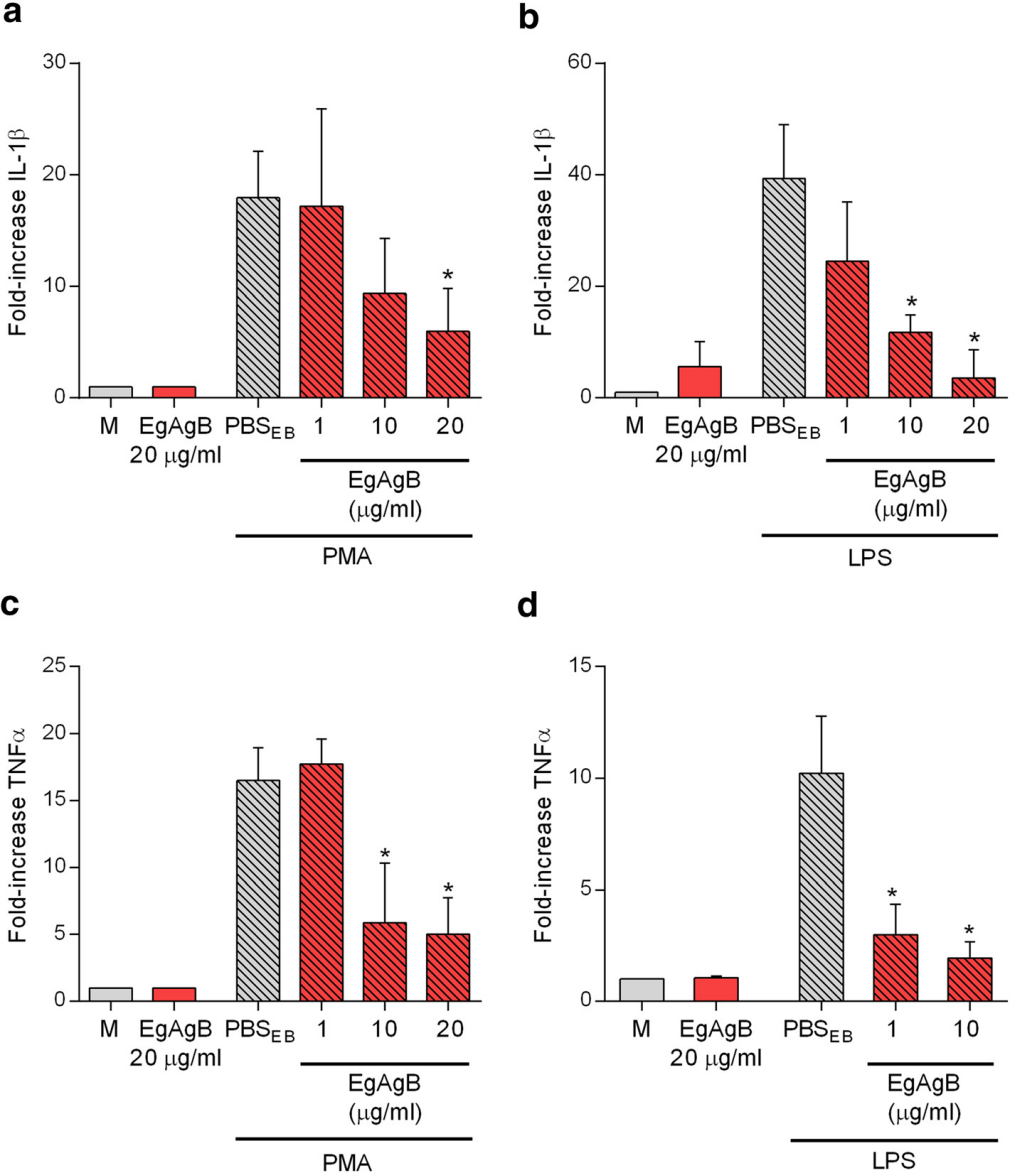
Effects of EgAgB on pro-inflammatory cytokine production by macrophages. THP-1 macrophages were stimulated with PMA (50 ng/ml) or LPS (0.1 ng/ml) for 12 h in the absence or presence of increasing concentrations of EgAgB. Secretion of IL-1β (**a**, **b**) and TNFα (**c**, **d**) were determined by ELISA in cell culture supernatant. IL-1β or TNFα levels are expressed as the fold-increase related to the basal secretion (normalised against the medium condition). Results correspond to mean values ± SEM of at least two independent experiments. Asterisks (*) indicate significant differences with respect to PMA or LPS-stimulated macrophages according to one-way ANOVA analysis followed by Tukey’s post-test (*p* < 0.05)

Studies on macrophage functional properties were performed using immunopurified EgAgB to eliminate trace levels of contaminants, particularly environmental pyrogens. The binding capacity of immunopurified EgAgB to monocytes and macrophages was similar to that observed for EgAgB preparations obtained by ultracentrifugation (Additional file 6). THP-1 macrophages were stimulated with PMA or LPS in the presence or absence of immunopurified EgAgB to assess whether EgAgB has the capacity to modulate inflammatory macrophage responses. Exposure to EgAgB on its own did not induce secretion of IL-1β or TNFα (Fig. 7). This result indicates that EgAgB does not activate macrophages in a similar way than PMA and LPS do. Furthermore, it confirmed that the EgAgB preparation exclude contamination by pyrogens. The presence of EgAgB during PMA or LPS stimulation caused a dose–response inhibition of IL-1β or TNFα secretion (Fig. 7), but no effects were observed on IL-10 production (Additional file 7). These results suggest that native EgAgB is able to trigger IL-10 independent-signalling pathways that control PKC and TLR4-mediated inflammatory responses. Inhibition of IL-1β or TNFα secretion by immunopurified EgAgB agrees with a previous report in which EgAgB was found to modulate the differentiation as well as the LPS induced-activation of immature dendritic cells [31]. In contrast with this report, in macrophages IL-10 secretion was not affected by exposure to EgAgB, suggesting signalling differences between cell types and/or EgAgB preparations. Activation of macrophages to an alternative-activated phenotype characterised by expression of arginase-I, was also investigated. For that purpose, BMDM were used since THP-1 macrophages are not appropriate to analyse arginase activity. BMDM were incubated with different doses of EgAgB for 24 h and arginase activity was determined in cell lysates. Since nitric oxide synthase 2 (NOS2) competes with arginase-I for their common substrate L-arginine, we examined NOS2 induction by measuring the production of nitrite in cell supernatants. In line with results obtained for cytokine secretion, EgAgB exposure to its own did not generate detectable levels of nitrite (lower than 3.125 μM), supporting that inflammatory pathways linked to NOS2 induction were not stimulated by EgAgB. On the other hand, in comparison with IL-4, exposure to EgAgB induced a slight increase in arginase activity (Additional file 8), suggesting that EgAgB is not a potent inducer of the alternative activation pathway in macrophages.

## Conclusions

The results we present in this work support the concept that once EgAgB reaches host tissues it may bind to tissue resident macrophages and inflammatory monocytes recruited during infection, through cell receptors likely involved in the recognition of plasma lipoproteins. EgAgB signalling through these receptors seems to induce a non-inflammatory phenotype in macrophages. In fact, this signalling could limit TLR4-mediated cytokine production that may occur during cyst growth, as a result of degradation of extracellular matrix [47]. Furthermore, it cannot be ruled out that EgAgB signalling would contribute to drive macrophage differentiation towards an alternative activation-like phenotype, which is consistent with the Th2-regulated immune response found in helminth infections, including echinococossis [40, 48]. Identification of cell receptor(s) and signalling pathways involved in EgAgB recognition by innate cells will be of foremost importance for understanding EgAgB role in parasite adaptation to host. Regarding EgAgB cell receptor(s), they likely recognise electrostatic motifs of EgAgB8/3 and EgAgB8/1, the latter constitutes the predominant protein component of native EgAgB present in HF. The fact that EgAgB cell receptors would also bind host HDL and LDL, together with the non-inflammatory phenotype observed after macrophage exposure to EgAgB, suggest a cross-talk between metabolic and inflammatory pathways [49], which requires further investigation.

## Additional files

Additional file 1:
**Comparison of the extent of biotinylation obtained for native EgAgB, rEgAgB8 subunits and OVA.** Proteins were adsorbed on ELISA microplates in a wide range of concentrations and the presence of biotin was determined using streptavidin-peroxidase and TMB/H_2_O_2_ for development. The absorbance at 450 nm was plotted vs. the protein concentration. (A) Comparison of the extent of biotinylation between OVA and native EgAgB; data are expressed as the mean values ± SEM of three analytical replicates B) Comparison of the extent of biotinylation between rEgAgB8 subunits; data are expressed as the mean values ± SEM of three analytical replicates. All preparations of biotinylated native EgAgB used in this work were controlled in respect to a preparation of OVA biotinylated in parallel. (TIF 6094 kb) Additional file 2:
**Analysis of the ability of EB7 mab to recognize rEgAgB8 subunits.** EB7 rEgAgB8/1, rEgAgB8/2 and rEgAgB8/3 were adsorbed on ELISA microplates to assess by ELISA the ability of Mab EB7 to recognize them. The binding of mab EB7 was developed using a peroxidase conjugated anti-mouse IgG followed by TMB/H_2_O_2_. The absorbance at 450 nm was plotted vs. the protein concentration. Data correspond to mean values ± SEM of analytical triplicates. As observed in the plot, mab EB7 recognises only EgAgB8/1. (TIF 62 kb) Additional file 3:
**Binding of native EgAgB to THP-1 derived monocytes and macrophages employing EB7 mab.** Binding of native EgAgB to THP-1 derived monocytes and macrophages was analysed employing EB7 mab followed by incubation with goat anti-mouse IgG/IgM antibody conjugated to FITC. As control, cells were incubated with binding buffer instead of native EgAgB (PBS), without adding EB7 mab (w/EB7) or using mouse IgG1 kappa isotype control instead of EB7 mab (IC). In left panels figure shows the histograms with the distribution of cell population as function of FITC fluorescence for controls (grey) and EgAgB-treated cells (red). Histograms are representative of four independent experiments for each cell type. In right panels binding indexes were plotted vs. native EgAgB concentrations (1, 5 and 10 μg/ml) for monocytes (upper panels) and macrophages (bottom panels). Data are expressed as mean ± SEM of four independent experiments. Asterisks (*) indicate significant differences with respect to the control with BB according to the analysis by *t*-test (*p* < 0.05), while number signs (#) indicate significant differences in the binding indexes obtained at 1 and 10 μg/ml native EgAgB (one-way ANOVA followed by Tukey’s post-test, *p* < 0.05). (TIF 11902 kb) Additional file 4:
**Binding of recombinant EgAgB8/1 to THP-1 derived monocytes and macrophages employing EB7 mab.** Binding of rEgAgB8/1 to THP-1 derived monocytes and macrophages was analysed employing EB7 mab followed by incubation with goat anti-mouse IgG/IgM antibody conjugated to FITC. As control, cells were incubated with binding buffer instead of native EgAgB (PBS), or in the absence of EB7 mab (w/EB7), or using mouse IgG1 kappa isotype control instead of EB7 mab (IC). Histograms (cell numbers vs. FITC fluorescence) corresponding to control (grey) and rEgAgB8/1-treated cells (blue) are shown on the left; they are representative of three independent experiments for each cell type. On the right, the binding indexes were plotted vs. rEgAgB8/1 concentration for monocytes (upper panel) and macrophages (bottom panel). Data are expressed as mean ± SEM of three independent experiments. Asterisks (*) indicate significant differences with respect to the control (PBS) according to the analysis by *t*-test (*p* < 0.05), while number signs (#) indicate significant differences in the binding indexes obtained at different native EgAgB concentrations (one-way ANOVA followed by Tukey’s post-test, *p* < 0.05). (TIF 12381 kb) Additional file 5:
**Involvement of plasma lipoprotein receptors in EgAgB binding to monocytes and macrophages.** (A) Cells were pre-incubated with ligands of LRP1 and SR-A receptors (lactoferrin and poly-inosinic acid [poli-I], respectively), centrifuged and then incubated with EgAgB in a fixed concentration (100 μg/ml). As control, BSA was used at the maximum lactoferrin and poli-I concentration employed. The binding index is expressed as mean ± SEM of analytical triplicates. (B) Binding analysis of native EgAgB to monocytes/macrophages F4/80^+^isolated from control (WT, empty bars) or LDLr^−/−^ mice (filled bars). Statistical analysis was undertaken by one-way ANOVA followed by Tukey’s post-test, no significant differences were observed. One representative experiment of two is shown in each panel. (TIF 302 kb) Additional file 6:
**Binding of immunopurified EgAgB to THP-1 derived monocytes and macrophages employing EB7 mab.** Binding of immunopurified EgAgB to THP-1 derived monocytes and macrophages was analysed employing EB7 mab followed by incubation with goat anti-mouse IgG/IgM antibody conjugated to FITC. As control, cells were incubated with binding buffer instead of native EgAgB (PBS), without adding EB7 mab (w/EB7) or using mouse IgG1 kappa isotype control instead of EB7 mab (IC). In left panels figure shows the histograms with the distribution of cell population as function of FITC fluorescence for controls (grey) and EgAgB-treated cells (red). Histograms are representative of three independent experiments for each cell type. In right panels binding indexes were plotted vs. native EgAgB concentrations (1 and 10 μg/ml) for monocytes (upper panels) and macrophages (bottom panels). Data are expressed as mean ± SEM of three independent experiments. Asterisks (*) indicate significant differences with respect to the control with BB according to the analysis by *t*-test (*p* < 0.05), while number signs (#) indicate significant differences in the binding indexes obtained at 1 and 10 μg/ml native EgAgB (one-way ANOVA followed by Tukey’s post-test, *p* < 0.05). (TIF 11689 kb) Additional file 7:
**Effects of EgAgB on IL-10 production by macrophages.** THP-1 macrophages were stimulated with LPS (0.1 ng/ml) for 12 h in absence or presence of increasing concentrations of EgAgB. Secretion of IL-10 was determined by ELISA in cell culture supernatant and expressed as the fold-increase related to the basal secretion (normalised against the medium condition). Results correspond to mean values ± SEM of two independent experiments. Number signs (#) indicate significant differences with respect to the medium condition according to one-way ANOVA analysis followed by Tukey’s post-test (*p* < 0.05). (TIF 24 kb) Additional file 8:
**Determination of arginase activity in bone marrow-derived macrophages stimulated with EgAgB.** Arginase activity was evaluated in BMDM incubated with PBS_EB_ (control), immunopurified EgAgB (0.2–20 μg/mL), or murine IL-4 (2.5 ng/ml) as a control of macrophage alternative activation. Arginase activity was expressed as mU per million of BMDM. The graph is representative of three independent experiments. Bars indicate SEM of analytical triplicates. Asterisks (*) indicate significant differences with respect to the control (medium) according to one-way ANOVA analysis followed by Tukey’s post-test (*p* < 0.05). (TIF 27 kb)

## Abbreviations

BB: Binding buffer (PBS containing 1 % (v/v) FS and 0.1 % NaN_3_)
BMDM: Bone marrow derived macrophages
EgAgB: Antigen B from *Echinococcus granulosus*
EgAgB_PLD-_: Control EgAgB for treatment with PLD
EgAgB_PLD+_: EgAgB treated with PLD
f_QS_: Q-Sepharose retained fraction
GL: Germinal layer
HDL: High-density lipoprotein
HF: Hydatid cyst fluid
HLBPs: Hydrophobic Ligand Binding Proteins
HPTLC: High performance thin layer chromatography
iBAQ: Intensity-based absolute quantification
LC-MS/MS: Liquid chromatography–mass spectrometry
LDL: Low-density lipoprotein
LDLr: LDL receptor
LPS: Lipopolysaccharides
LRP1: LDL receptor related protein 1
MALDI-TOF/TOF: Matrix-assisted laser desorption/ionization time-of-flight
OVA: Ovalbumin
PBS_EB_: PBS containing 5 mM EDTA and 20 μM BHT
PC: Phosphatidylcholine
PC/PS-LUVs: Large unilamellar vesicles composed of PC and PS (50:50 mol:mol)
PC-LUVs: Large unilamellar vesicles composed of PC
PLD: Phospholipase D
PS: Phosphatidylserine
rEgAgB8: recombinant lipid-free EgAgB8 subunits
RP-HPLC: Reversed-phase high performance liquid chromatography
SR-A: Class A scavenger receptor
