# Supplementary figures and images for: Echinococcus granulosus Antigen B binds to monocytes and macrophages modulating cell response to inflammation
Source: Parasit Vectors. 2016 Feb 4;9:69. doi: 10.1186/s13071-016-1350-7 (PMC4743400; doi:10.1186/s13071-016-1350-7)

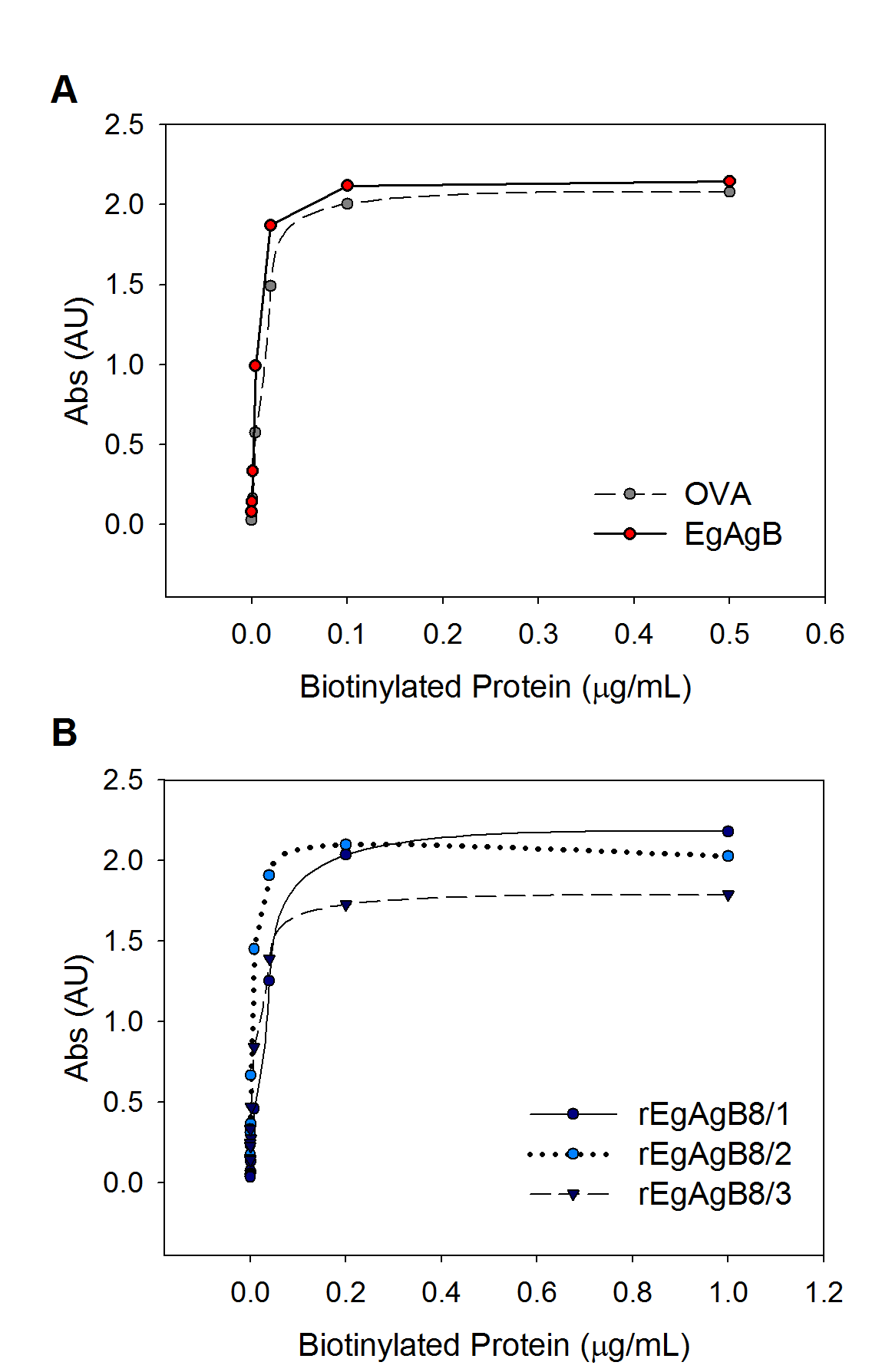

Supplement: Additional file 1: — Comparison of the extent of biotinylation obtained for native EgAgB, rEgAgB8 subunits and OVA. Proteins were adsorbed on ELISA microplates in a wide range of concentrations and the presence of biotin was determined using streptavidin-peroxidase and TMB/H2O2 for development. The absorbance at 450 nm was plotted vs. the protein concentration. (A) Comparison of the extent of biotinylation between OVA and native EgAgB; data are expressed as the mean values ± SEM of three analytical replicates B) Comparison of the extent of biotinylation between rEgAgB8 subunits; data are expressed as the mean values ± SEM of three analytical replicates. All preparations of biotinylated native EgAgB used in this work were controlled in respect to a preparation of OVA biotinylated in parallel. (TIF 6094 kb) [file 13071_2016_1350_MOESM1_ESM.tif]

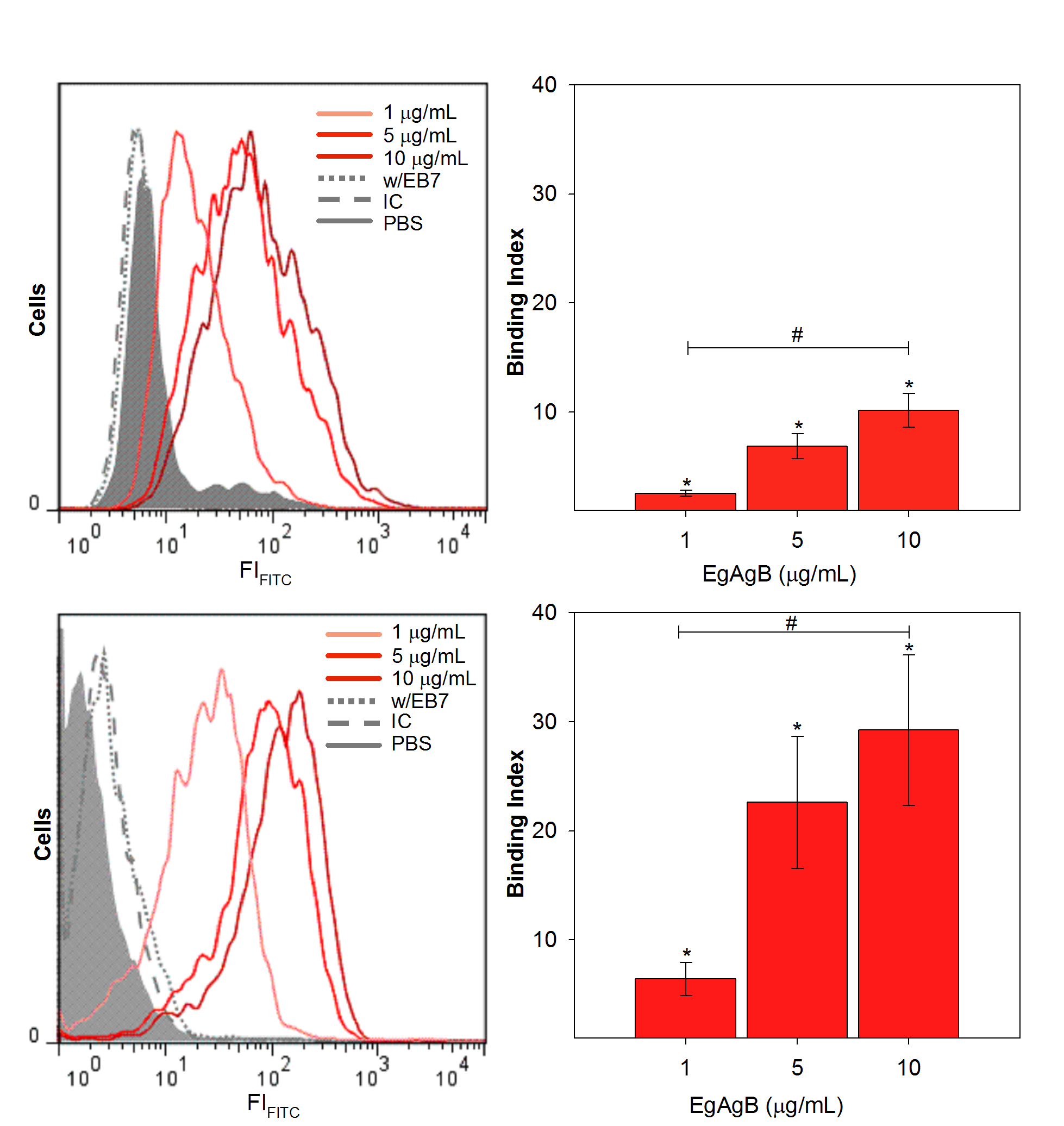

Supplement: Additional file 3: — Binding of native EgAgB to THP-1 derived monocytes and macrophages employing EB7 mab. Binding of native EgAgB to THP-1 derived monocytes and macrophages was analysed employing EB7 mab followed by incubation with goat anti-mouse IgG/IgM antibody conjugated to FITC. As control, cells were incubated with binding buffer instead of native EgAgB (PBS), without adding EB7 mab (w/EB7) or using mouse IgG1 kappa isotype control instead of EB7 mab (IC). In left panels figure shows the histograms with the distribution of cell population as function of FITC fluorescence for controls (grey) and EgAgB-treated cells (red). Histograms are representative of four independent experiments for each cell type. In right panels binding indexes were plotted vs. native EgAgB concentrations (1, 5 and 10 μg/ml) for monocytes (upper panels) and macrophages (bottom panels). Data are expressed as mean ± SEM of four independent experiments. Asterisks (*) indicate significant differences with respect to the control with BB according to the analysis by t-test (p < 0.05), while number signs (#) indicate significant differences in the binding indexes obtained at 1 and 10 μg/ml native EgAgB (one-way ANOVA followed by Tukey’s post-test, p < 0.05). (TIF 11902 kb) [file 13071_2016_1350_MOESM3_ESM.tif]

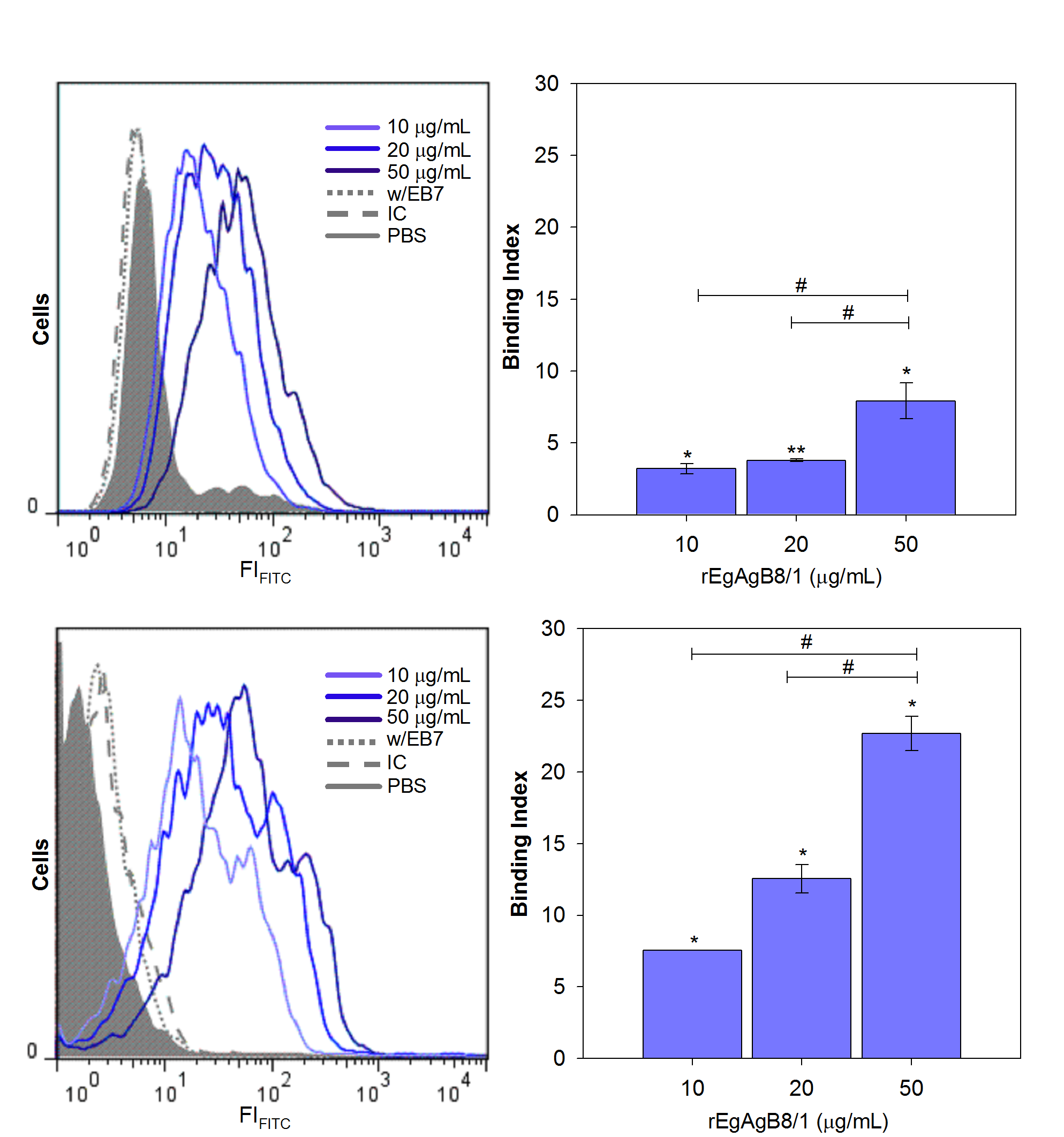

Supplement: Additional file 4: — Binding of recombinant EgAgB8/1 to THP-1 derived monocytes and macrophages employing EB7 mab. Binding of rEgAgB8/1 to THP-1 derived monocytes and macrophages was analysed employing EB7 mab followed by incubation with goat anti-mouse IgG/IgM antibody conjugated to FITC. As control, cells were incubated with binding buffer instead of native EgAgB (PBS), or in the absence of EB7 mab (w/EB7), or using mouse IgG1 kappa isotype control instead of EB7 mab (IC). Histograms (cell numbers vs. FITC fluorescence) corresponding to control (grey) and rEgAgB8/1-treated cells (blue) are shown on the left; they are representative of three independent experiments for each cell type. On the right, the binding indexes were plotted vs. rEgAgB8/1 concentration for monocytes (upper panel) and macrophages (bottom panel). Data are expressed as mean ± SEM of three independent experiments. Asterisks (*) indicate significant differences with respect to the control (PBS) according to the analysis by t-test (p < 0.05), while number signs (#) indicate significant differences in the binding indexes obtained at different native EgAgB concentrations (one-way ANOVA followed by Tukey’s post-test, p < 0.05). (TIF 12381 kb) [file 13071_2016_1350_MOESM4_ESM.tif]

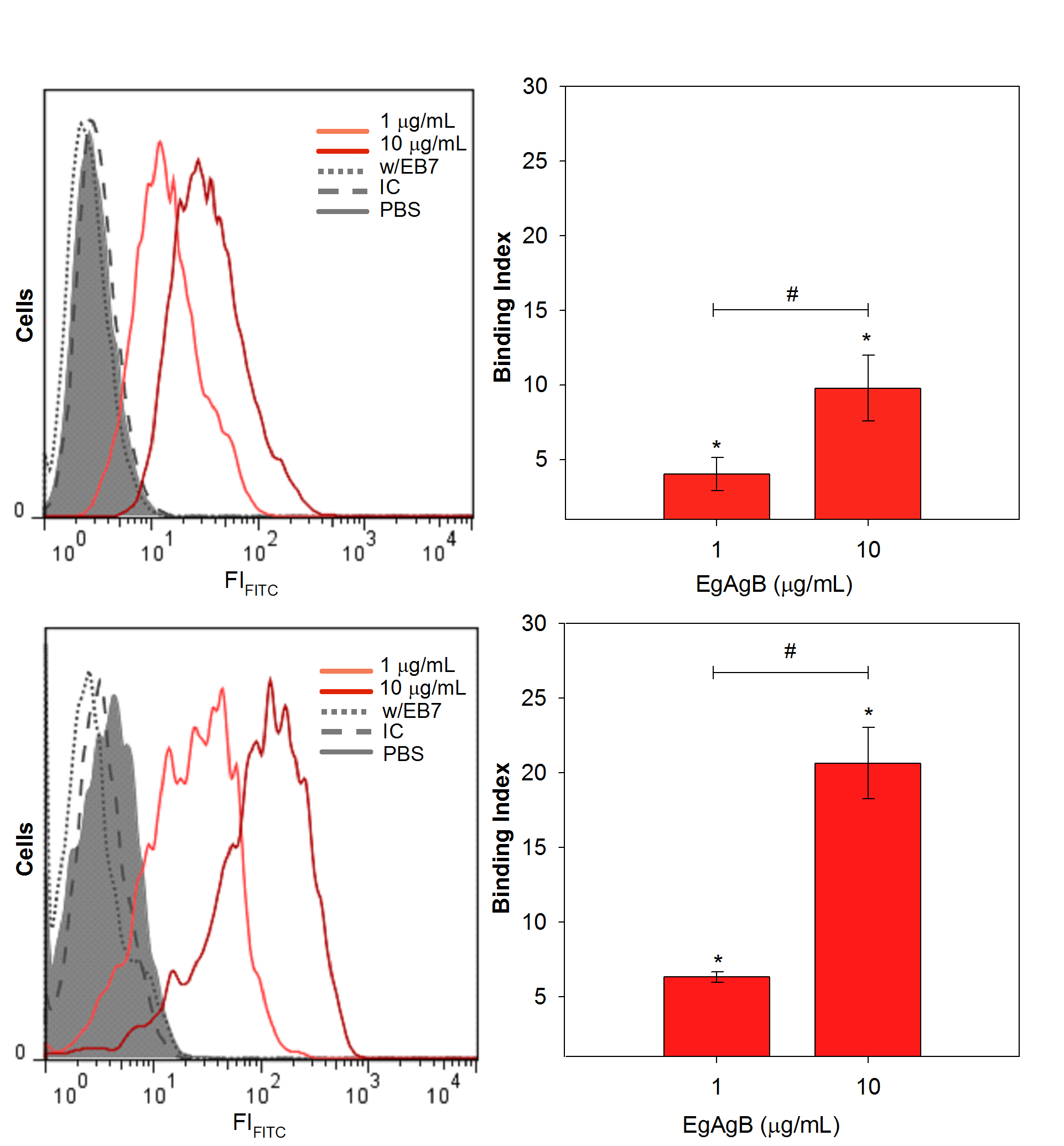

Supplement: Additional file 6: — Binding of immunopurified EgAgB to THP-1 derived monocytes and macrophages employing EB7 mab. Binding of immunopurified EgAgB to THP-1 derived monocytes and macrophages was analysed employing EB7 mab followed by incubation with goat anti-mouse IgG/IgM antibody conjugated to FITC. As control, cells were incubated with binding buffer instead of native EgAgB (PBS), without adding EB7 mab (w/EB7) or using mouse IgG1 kappa isotype control instead of EB7 mab (IC). In left panels figure shows the histograms with the distribution of cell population as function of FITC fluorescence for controls (grey) and EgAgB-treated cells (red). Histograms are representative of three independent experiments for each cell type. In right panels binding indexes were plotted vs. native EgAgB concentrations (1 and 10 μg/ml) for monocytes (upper panels) and macrophages (bottom panels). Data are expressed as mean ± SEM of three independent experiments. Asterisks (*) indicate significant differences with respect to the control with BB according to the analysis by t-test (p < 0.05), while number signs (#) indicate significant differences in the binding indexes obtained at 1 and 10 μg/ml native EgAgB (one-way ANOVA followed by Tukey’s post-test, p < 0.05). (TIF 11689 kb) [file 13071_2016_1350_MOESM6_ESM.tif]
